# Three immunizations with Novavax’s protein vaccines increase antibody breadth and provide durable protection from SARS-CoV-2

**DOI:** 10.1038/s41541-024-00806-2

**Published:** 2024-01-20

**Authors:** Klara Lenart, Rodrigo Arcoverde Cerveira, Fredrika Hellgren, Sebastian Ols, Daniel J. Sheward, Changil Kim, Alberto Cagigi, Matthew Gagne, Brandon Davis, Daritza Germosen, Vicky Roy, Galit Alter, Hélène Letscher, Jérôme Van Wassenhove, Wesley Gros, Anne-Sophie Gallouët, Roger Le Grand, Harry Kleanthous, Mimi Guebre-Xabier, Ben Murrell, Nita Patel, Gregory Glenn, Gale Smith, Karin Loré

**Affiliations:** 1 Department of Medicine Solna, Division of Immunology and Allergy, Karolinska Institutet, Stockholm, Sweden; 2 Karolinska University Hospital, Stockholm, Sweden; 3 Center for Molecular Medicine, Karolinska Institutet, Stockholm, Sweden; 4 Department of Microbiology, Tumor and Cell Biology, Karolinska Institutet, Stockholm, Sweden; 5 Vaccine Research Center, National Institute of Allergy and Infectious Diseases, National Institutes of Health, Bethesda, MD USA; 6 Ragon Institute of MGH, MIT, and Harvard, Cambridge, MA USA; 7 Université Paris-Saclay, Inserm, CEA, Center for Immunology of Viral, Auto-immune, Hematological and Bacterial diseases (IMVA-HB/IDMIT), Fontenay-aux-Roses & Le Kremlin-Bicêtre, Paris, France; 8 Bill & Melinda Gates Foundation, Seattle, WA USA; 9 Novavax Inc, Gaithersburg, MD USA; 10 Present Address: SK Biosciences, Boston, MA USA

**Keywords:** Protein vaccines, Immunological memory, Antibodies, Viral infection

## Abstract

The immune responses to Novavax’s licensed NVX-CoV2373 nanoparticle Spike protein vaccine against SARS-CoV-2 remain incompletely understood. Here, we show in rhesus macaques that immunization with Matrix-M^TM^ adjuvanted vaccines predominantly elicits immune events in local tissues with little spillover to the periphery. A third dose of an updated vaccine based on the Gamma (P.1) variant 7 months after two immunizations with licensed NVX-CoV2373 resulted in significant enhancement of anti-spike antibody titers and antibody breadth including neutralization of forward drift Omicron variants. The third immunization expanded the Spike-specific memory B cell pool, induced significant somatic hypermutation, and increased serum antibody avidity, indicating considerable affinity maturation. Seven months after immunization, vaccinated animals controlled infection by either WA-1 or P.1 strain, mediated by rapid anamnestic antibody and T cell responses in the lungs. In conclusion, a third immunization with an adjuvanted, low-dose recombinant protein vaccine significantly improved the quality of B cell responses, enhanced antibody breadth, and provided durable protection against SARS-CoV-2 challenge.

## Introduction

Protein subunit vaccines have a historical record of favorable safety profiles. Novavax’s protein subunit vaccine NVX-CoV2373, containing prefusion-stabilized Spike protein (BV2373) and saponin-based Matrix-M^TM^ adjuvant^1^, was the first protein vaccine platform to be authorized against COVID-19 after demonstrating 89.7% efficacy against SARS-SoV-2 infection in phase III clinical trials^2,3^, on par with licensed mRNA vaccines^4,5^. However, high immunogenicity of mRNA vaccines induces systemic immune perturbations through interferon-related pathways, changes in the composition of circulating immune cells, and pro-inflammatory cytokines in the serum^6–9^. Fewer NVX-CoV2373 vaccinees reported fever after the second dose compared to individuals receiving mRNA-1273 or BNT162b2^2,4,5^.

Vaccines with saponin-containing adjuvants, including Matrix-M^TM^, have shown superior antibody responses compared to conventional adjuvants like Alum^10,11^, and have been tested in preclinical and clinical studies with vaccines against HIV^10,12,13^, Ebola^11^, influenza^14,15^, malaria^16^ and SARS-CoV-2^2,17–19^. Matrix-M^TM^ was shown to promote infiltration of monocytes and neutrophils into the site of injection^12^ and enhance trafficking of immune cells into secondary lymphoid organs after immunization^20,21^, partly in a histamine-dependent manner^20^. Experiments in small animal models suggest that free saponins disrupt lysosomal membranes in antigen-presenting cells (APCs) after uptake which promotes activation of NLRP3 inflammasome, leading to production of pro-inflammatory cytokines. This milieu induces secretion of IFNγ by natural killer (NK) cells in the draining lymph nodes (dLNs), supporting development of Th1-polarized T cell responses^22^. However, systemic immunological mechanisms exerted by Matrix-M^TM^ remain underexplored.

Adjuvanted protein subunit vaccines against COVID-19 elicit high levels of neutralizing antibodies and protect non-human primates (NHPs) from SARS-CoV-2 infection^17,23–26^. Nevertheless, protection was usually assessed 2–4 weeks after the second immunization, coinciding with the peak of the immune response. More knowledge on long-term protection is essential to optimize the frequency of boost immunizations. A sustained protective effect against SARS-CoV-2 challenge was demonstrated 12 and 6 months after mRNA-1273^27^ and I53-50-RBD protein nanoparticle^28^ immunization in NHPs, respectively. When infected several months after last vaccination, immunized animals displayed significant, although delayed and incomplete protection in the lungs compared to challenging early after vaccination, which was likely mediated by the recall responses in the mucosal tissues^23,27,29^.

After SARS-CoV-2 infection or immunization, most individuals develop potent immune responses to the Spike (S) protein and its receptor binding domain (RBD)^30,31^. RBD is the primary target of neutralizing antibodies^32,33^ and thus under heavy evolutionary pressure, especially at the receptor-binding site^34,35^. Elicitation of broadly neutralizing B cell responses is therefore critical for developing highly efficacious vaccine immunity to past, present, and future SARS-CoV-2 variants^36,37^. Several broadly neutralizing antibodies specific for non-RBD epitopes have been described in refs. ^38–40^, but their representation in the serum is generally rare. Nevertheless, a considerable proportion (>50%) of S-specific B cells bind non-RBD epitopes^30,41–44^, and non-neutralizing antibodies can contribute to protection through fragment crystallizable region (Fc)-mediated antibody functions^45,46^. Overall, the temporal development of the S- and RBD-specific B cells has not been fully resolved, especially in the early phases of vaccine-induced immunity. Studies in mice showed that the memory B cells, formed in the primary response to the antigen, predominantly contribute to the serum antibody pool upon secondary exposure to a homologous antigen^47^, while the same circulating antibodies influence the recruitment of naïve B cells to the germinal center reaction upon re-immunization^48^. Together, this underscores the importance of understanding immunological events after priming immunizations and their long-term implications.

Here, we investigated multiple aspects of the immune responses to NVX-CoV2373 as well as the effect of a booster dose with an experimental vaccine NVX-CoV2443 based on the variant of concern (VOC) P.1 in NHPs. We profiled the early innate events upon Matrix-M^TM^ immunization, showing that the innate events are highly restricted to the site of injection and the dLNs, with little systemic spillover. Furthermore, through a detailed evaluation of the humoral and cellular immune responses, we show that although RBD is intrinsically immunodominant in the initial immune response, B cell specificity is skewed towards non-RBD epitopes with each subsequent exposure to the S protein. This effect is, at least partially, mediated by circulating antibodies. Finally, using a high dose challenge model we show significant protection against infection seven months after the final vaccination compared to controls, with a clear correlation between higher antibody levels at the time of challenge and reduced viral loads in the upper and lower respiratory tract. Our findings are relevant for clinical vaccine development, contributing to a deeper understanding of immune responses upon Matrix-M^TM^ adjuvant administration as well as the specificity of the B cell responses after immunization with recombinant SARS-CoV-2 S protein.

## Results

### Limited systemic innate immune activation after Matrix-M^TM^ immunization

**Fig. 1:**
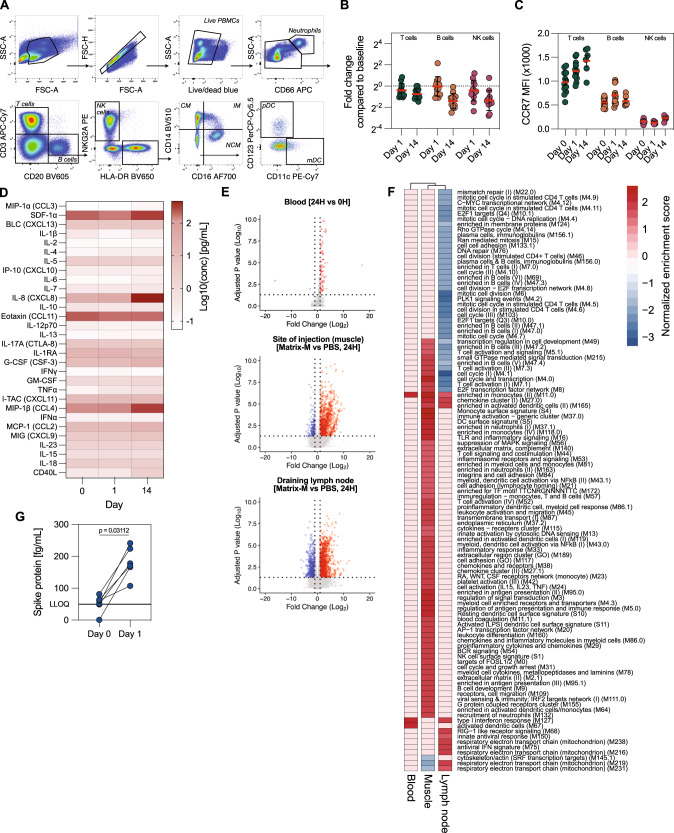
Limited systemic innate activation after Matrix-M^TM^ immunization. **A** Gating strategy for immunophenotyping of PBMC samples. **B** Fold change in frequency of different lymphocytes subsets 1 and 14 days after first immunization compared to day 0 (*n* = 12). **C** CCR7 expression on different lymphocytes subsets on days 0, 1, and 14 after first immunization (*n* = 6–12). **D** Serum cytokine levels on days 0, 1 and 14 after first immunization (*n* = 6). **E** Differentially expressed genes (DEGs) in blood, at the site of injection and in the draining lymph node 24 h after first immunization with NVX-CoV2373 (50 μg Matrix-M^TM^, blood) or Matrix-M^TM^ alone (75 μg, site of injection and draining lymph node) (Blood: *n* = 12, Muscle and dLN: *n* = 3-4). **F** Gene set enrichment analysis of DEGs using blood transcription modules in different tissues. **G** Detection of soluble Spike protein in circulation at days 0 and 1 after first immunization (*n* = 6). LLOQ = lower limit of quantification. Data is presented as geometric mean ± geometric SD (**B**) or mean ± SEM (**C**). The dotted line represents fold change of 1 (i.e., no change from baseline) (**B**) or lower limit of quantification (**G**). Statistical analysis was performed using Wilcoxon test (**G**).

Although Matrix-M^TM^ has been shown to be a highly potent adjuvant^2,12,16,19^, innate immune responses to Matrix-M^TM^ immunizations are incompletely understood. We profiled the changes induced by Matrix-M^TM^ in plasma and PBMCs using Luminex assays, flow cytometry, and RNA sequencing. Rhesus macaques were immunized with a clinical dose of NVX-CoV2373, containing 5 μg prefusion-stabilized ancestral S protein and 50 μg Matrix-M^TM^, and innate immune responses were analyzed on days 0, 1, and 14. Most clinical chemistry and hematology parameters were not affected by immunization, and some showed transient changes at 24 h (Supplementary Fig. 1A, B). The changes in composition of circulating PBMCs were analyzed by combining complete blood counts (Supplementary Fig. 1A) with flow cytometry data (Fig. 1A). Intermediate monocytes and plasmacytoid dendritic cells were transiently increased 24 h after immunization, while non-classical monocytes showed an increase in numbers 14 days after immunization (Supplementary Fig. 2A, B). In contrast, numbers of lymphocytes, specifically T and NK cells, were considerably decreased both 1 and 14 days after immunization compared to baseline (Fig. 1B). This decrease coincided with increased CCR7 expression on circulating lymphocytes (Fig. 1C), suggesting a potential for increased migration of lymphocytes to the lymphoid organs even 14 days after vaccination.

Contrary to reports following mRNA vaccination, no plasma cytokines were elevated 24 h after prime **(**Fig. 1D). However, several cytokines, such as CXCL12, CCL3, CCL4, and IL-8 were elevated 14 days after immunization (Supplementary Fig. 2C). The systemic cytokine levels may reflect the responses occurring locally at the immunization site. Using RNA sequencing, we measured the transcriptomic profiles in blood and biopsies from the site of injection and dLNs. This showed a clear distinction between local and systemic immune responses to Matrix-M^TM^ administration. Limited transcriptional changes were detected in the blood 24 h after immunization (Fig. 1E), which were mainly associated with myeloid cells and type I interferon responses (Fig. 1F). At the site of injection (muscle) and in dLNs, we observed larger transcriptomic differences between Matrix-M^TM^ or PBS-injected tissues (Fig. 1E**)**, showing that immune activation was restricted to the local sites. The upregulated immune modules in Matrix-M^TM^-injected muscle were associated with cytokine production, myeloid cells, neutrophils, cell activation, and phagocytosis (Fig. 1F), likely reflecting the infiltration of innate immune cells to the injection site as we previously reported^12,49^. Cell cycle and lymphocyte-associated modules were downregulated in the dLNs 24 h after immunization (Fig. 1F). Lastly, S protein has been detected in plasma in the range of 10^3^-10^5^ fg/mL after mRNA vaccine administration^50,51^. Using the same assay, we detected S in the plasma in the range of 100–300 fg/mL after immunization with NVX-CoV2373, representing a 10- to 333-fold lower concentration compared to that upon immunization with mRNA vaccines (Fig. 1G). This may further indicate that distribution of NVX-CoV2373 is restricted with limited systemic dissemination.

### High immunogenicity of Novavax’s protein subunit vaccine

**Fig. 2:**
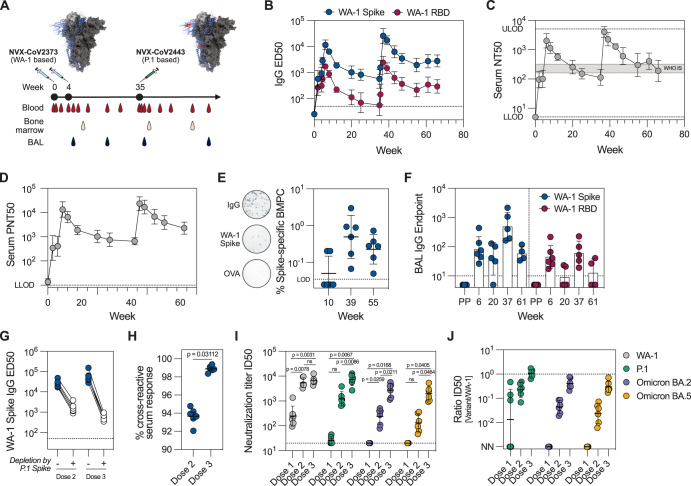
Increased breadth after the heterologous boost immunization. **A** Study design and sampling schedule. Animals were immunized with NVX-CoV2373 at weeks 0 and 4 and boosted with NVX-CoV2443 at week 35. WA-1 Spike structure was obtained from PBD ID J771, with mutations in P.1 Spike compared to WA-1 Spike labeled in red. **B** IgG plasma binding titers to WA-1 Spike and RBD (*n* = 6). **C** Serum neutralization of WA-1/2020 SARS-CoV-2 virus (*n* = 6). Gray shaded area represents the NT50 of WHO international standard NIBSC 20/136 (WHO IS). **D** Serum neutralization using WA-1 Spike pseudotyped VSV virus particles (*n* = 6). **E** Frequency of WA-1 Spike-specific bone marrow plasma cells (BMPC) (*n* = 6). Representative ELISpot wells are shown on the left. All data is background subtracted based on OVA wells. **F** IgG binding antibodies in BAL (*n* = 4–6. Samples with total IgG endpoint titer <1000 were excluded from analysis). PP = pre-pandemic BAL samples. **G**, **H** Cross-reactive serum antibodies to WA-1 and P.1 Spike protein (*n* = 6). IgG binding titers to WA-1 Spike with and without depletion by P.1 Spike (**G**) and proportion of depleted antibodies (**H**). Serum neutralization of pseudotyped VSV viruses carrying variant Spike proteins (P.1, Omicron BA.2 and BA.5) at different timepoints (**I**) and the ratios between neutralization of the variants compared to WA-1 (**J**) (*n* = 6). NN = not neutralizing. Data is presented as geometric mean ± geometric SD (**B**–**F**, **I**, **J**) or mean ± SEM (**H**). Statistical analysis was performed using Wilcoxon test (**H**) and repeated measures two-way ANOVA with the Geisser-Greenhouse correction and Tukey’s multiple comparisons test (**I**). Dose 1, dose 2, and dose 3 refer to 2 weeks after each immunization. Dotted line represents lower level of detection (**B**, **D**–**G**, **I**) or the ratio of 1 (**J**). LLOD lower level of detection, ULOD upper level of detection.

Clinical trials have demonstrated the efficacy of NVX-CoV2373^2,3^, but the adaptive immune responses to the vaccine have not been characterized in detail. Six rhesus macaques were immunized with NVX-CoV2373 at weeks 0 and 4. The animals were boosted with NVX-CoV2443, a variant COVID-19 vaccine based on the P.1 (Gamma) S seven months after dose 2, at week 35. Blood, bone marrow and bronchoalveolar lavage (BAL) were frequently sampled during the study period (Fig. 2A). Two weeks after the first immunization, all animals developed detectable S-binding IgG (Fig. 2B) and neutralizing antibodies in the serum (Fig. 2C, D). The second immunization significantly amplified the response, with neutralizing titers exceeding WHO international standard by ~10-fold. The third immunization with variant S antigen boosted the systemic antibody responses above the levels measured after the second dose. S-specific bone marrow resident plasma cells were detected in all animals both at weeks 39 and 55 (Fig. 2E), suggesting that a durable plasma cell pool was established after three immunizations. The mucosal antibodies in the lungs, quantified as S-binding IgG antibody titers in the BAL fluid, were detectable (Fig. 2F) after two immunizations (week 6) and further boosted by the third dose (week 37), resembling the kinetics of serum antibodies.

Apart from mucosal immunity, long-term vaccine-mediated protection likely depends on the induction of cross-reactive antibody responses capable of neutralizing current and future SARS-CoV-2 variants. We measured the proportion of WA-1/P.1 cross-reactive antibodies in the sera through competition ELISA assays, where all P.1 cross-reactive IgG was depleted by excess P.1 S in solution (Fig. 2G). WA-1 and P.1 S proteins differ by 12 amino acid residues (0.9% of total protein sequence), and yet only 94% of plasma antibodies were able to cross-bind after the second vaccination. The third, heterologous immunization, enhanced the cross-binding of plasma antibodies to about 99% (Fig. 2H), indicating that heterologous immunization can increase the breadth of B cell responses. Neutralization of pseudoviruses carrying S proteins of different SARS-CoV-2 VOCs showed that after the first dose, the serum antibodies could only mediate neutralization of WA-1 pseudoviruses, but after the second and third dose the neutralization breadth was substantially enhanced. Following the third dose, P.1 neutralization titer was equal to WA-1, and neutralization of highly immune-evasive BA.2 and BA.5 Omicron strains was only 2.2- and 3.0-fold lower compared to WA-1, respectively (Fig. 2I, J).

Furthermore, functional characterization of the vaccine-elicited antibodies was performed against different coronavirus S and RBD proteins. Antibody-dependent cellular phagocytosis (ADCP), antibody-dependent neutrophil phagocytosis (ADNP) and antibody-dependent complement deposition (ADCD) using WA-1, P.1 and Omicron Spike as antigens showed a delayed response towards Omicron as compared to WA-1 or P.1 (Supplementary Fig. 3A–C). The delayed kinetics for Omicron-derived antigens were also observed for different antigen-specific antibody isotypes and subclasses (Supplementary Fig. 3D–F) and their ability to bind Fc receptors (Supplementary Fig. 3G–I). A principal component (PC) analysis of all antibody features distinctly separated the different timepoints, with week 6 and 37 showing the most variation across Dim1 (Supplementary Fig. 3J). This separation was overall driven by IgG, IgA and FcR3A binding (Supplementary Fig. 3K). Some separation was also observed against Dim2, with ADCP against the vaccine antigen and binding to FcR2A-4 of Omicron RBD-specific IgG as the highest contributors (Supplementary Fig. 3K). A minimal set of selected features used for co-correlate analysis indicated that FcR-binding, antibody isotypes and subclasses, and Fc effector functions had a strong positive correlation between different humoral parameters (Supplementary Fig. 3L).

S-specific CD4 and CD8 T cell responses in blood and BAL (Supplementary Fig. 4A, B) showed that the CD4 T cell response was readily detectable and highly Th1 polarized (Supplementary Fig. 4C), while S-specific CD8 T cells were low (Supplementary Fig. 4C). T cell responses displayed similar kinetics as the humoral response, with the peak Th1 frequencies detected 2 weeks after each boost (Supplementary Fig. 4D).

**Fig. 3:**
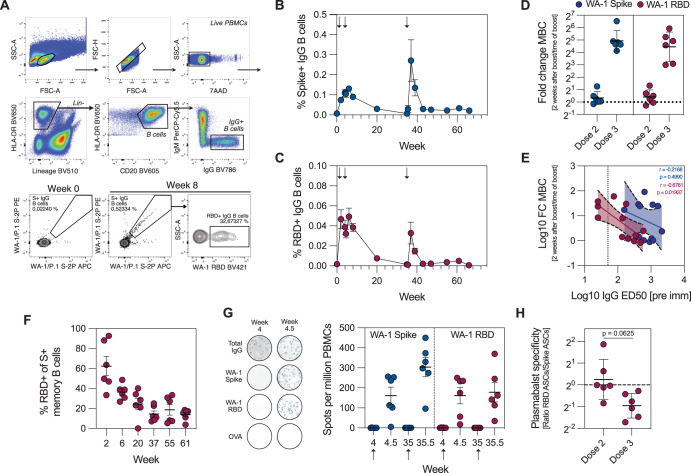
Shift in RBD immunodominance with each immunization. **A** Gating strategy for identification of Spike- and RBD-specific MBCs. Lineage channel contains CD3, CD11c, CD14, CD16, and CD123. Frequency of Spike- (**B**) and RBD-specific MBCs (**C**) (*n* = 6). **D** Increase in frequency of Spike- and RBD-specific MBCs 2 weeks after boost 1 and 2 compared to the frequency at the time of boost (*n* = 6). **E** The correlation between pre-existing antibody titers and the increase in MBC frequency after boost immunization (*n* = 6). **F** Proportion of RBD-binding MBCs out of all S-binding MBCs (*n* = 6). **G** Plasmablast responses in blood (*n* = 6). Representative ELISpot wells are shown on the left. All data is background subtracted based on OVA wells. **H** Ratio between Spike and RBD-specific plasmablasts after each boost (*n* = 6). Data is presented as mean ± SEM (**B**, **C**, **F**, **G**) or geometric mean ± geometric SD (**D**, **H**). Statistical analysis was performed using Spearman correlation (**E**) or Wilcoxon test (**H**). Boost 1 and boost 2 refer to 2 weeks after boost 1 and 2, respectively. Dotted line corresponds to fold change = 1, i.e., no change (**D**, **H**) or the lower limit of detection (**E**). Arrows indicate immunizations.

S- and RBD-specific memory B cells (MBCs) in blood were detectable 2 weeks after the first immunization, with a further minor increase after the second dose. S-specific MBCs waned over the 7-month follow-up period, when they were again boosted by the third, variant vaccine dose (Fig. 3A–C). The third immunization induced a higher fold-increase of S- and RBD-specific MBCs compared to the second dose (Fig. 3D). The fold increases for RBD-specific MBCs, but not S-specific MBCs, were negatively correlated with pre-existing IgG antibody titers (*r* = −0.6761, *p* = 0.01937) (Fig. 3E). A progressive decrease in the relative frequency of RBD-specific MBCs over 61 weeks after the prime immunization (Fig. 3F) suggests that, in line with recent reports^48^, the specificity of circulating antibodies can influence secondary B cell responses. Finally, enumeration of short-lived plasmablasts after the boost immunizations showed a relative decrease in RBD-specific plasmablasts after the third immunization (Fig. 3G, H), in agreement with the MBC data. Together, these data suggest marked immunodominance of RBD-specific B cell responses after primary immunization with NVX-CoV2373, which are skewed towards non-RBD epitopes with each subsequent dose.

### Shift in RBD immunodominance

**Fig. 4:**
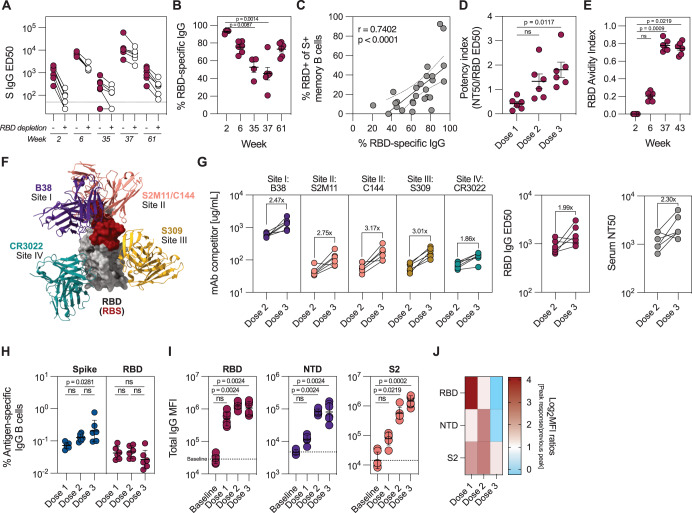
Expansion of non-RBD-specific B cell responses following boost immunizations. WA-1 Spike-binding IgG titers in plasma before and after depletion of RBD-binding antibodies (**A**), and proportion of RBD-binding antibodies at different timepoints (**B**) (*n* = 6). **C** Correlation between proportion of RBD-specific IgG antibodies and RBD-specific MBCs in the blood (*n* = 6). **D** Neutralization potency index, defined as the ratio between WA-1 neutralizing and RBD-binding antibody titers 2 weeks after each immunization (*n* = 6). **E** Avidity index of RBD-binding IgG antibodies at different timepoints (*n* = 6), determined by chaotropic wash ELISA with 2 M NaSCN as the chaotropic reagent. **F** Schematic of RBD with representative antibodies for four defined binding classes (PDB IDs 7K90 (RBD + C144 mAb), 7BZ5 (B38 mAb), 6WPS (S309 mAb), 6W41 (CR3022 mAb)). Receptor binding site (RBS) is labeled in red. **G** Relative serum reactivity to each of the four defined RBD-binding antibody classes (site I-site IV, *n* = 6), determined through ELISA competition assay using biotinylated monoclonal antibodies listed in (**F**). Total RBD-binding IgG and neutralization titers are shown on the right. Dose 2 and 3 represent timepoints 4 weeks after each immunization. **H** Frequency of Spike- and RBD-specific MBCs 2 weeks after each immunization (*n* = 6). Binding to different Spike subdomains (RBD, NTD, or S2) at selected timepoints, measured by multiplexed Luminex bead-based assay (*n* = 6) (**I**) and the fold changes in the binding titers from timepoint to timepoint (Dose 1 = week 2/week 0, Dose 2 = week 6/week 2, Dose 3 = week 37/week 6) (**J**). Data is presented as mean ± SEM (**B**, **D**, **E**) or geometric mean ± geometric SD (**H**, **I**). Statistical analysis was performed using Kruskal-Wallis test with Dunn’s post hoc correction (**B**), Spearman correlation (**C**) or Friedman’s test with Dunn’s post hoc correction (**D**, **E**, **H**, **I**). Baseline refers to week 0, dose 1, dose 2 and dose 3 refer to 2 weeks after each immunization in all panels except in (**G**). Dotted line corresponds to lower level of detection (**A**), fold change = 1 (**D**, **H**). or the baseline signal at week 0 (**I**).

The skewing of the S-specific MBCs towards non-RBD epitopes with sequential immunizations prompted us to delve deeper into the specificities of serum antibody response. To assess the proportion of RBD-specific IgG antibodies in the serum, we developed a competition ELISA to evaluate the proportion of RBD-specific IgG by sequestering RBD-specific antibodies with excess RBD in solution. The relative proportion of RBD-reactive IgG was calculated from the decrease in ED_50_ values (Fig. 4A). We found a significant decrease in the proportion of RBD-specific IgG between week 2 and 37, corresponding to post-first and post-third dose, respectively (Fig. 4B). While the majority (average 92.6%, range 89.8–93.8%) of S-specific serum antibodies after dose 1 bound RBD, only 45.4% (range 20.9–75.3%) were RBD-specific after the third dose. At week 61, the proportion of RBD-specific serum antibodies increased to an average of 73.1% (range 62.2–81.0%). This shift may represent the differential specificity distribution between short-lived plasmablasts and long-live plasma cells as main antibody-secreting populations at weeks 37 and 61, respectively. Moreover, proportions of RBD-specific plasma IgG and circulating MBCs were positively correlated (*r* = 0.7402, *p* < 0.0001) (Fig. 4C). Despite the decrease in proportion of RBD-reactive MBCs, the antibody potency, defined as the ratio between neutralizing and binding antibody titers, increased with each immunization (Fig. 4D). Affinity maturation of RBD-specific MBCs, displayed as increased avidity of RBD-specific IgG (Fig. 4E), likely contributed to increased antibody potency.

Quantification of responses to different RBD epitopes through a competition ELISA assay with well-characterized biotinylated monoclonal antibodies^52^ (Fig. 4F) revealed a 2–3-fold increase in antibody response to all four major RBD epitopes after dose 3 (Fig. 4G). Responses to different sites were boosted to a similar extent and corresponded to an overall increase in RBD-binding and neutralizing antibodies at these timepoints (Fig. 4G), indicating that the anti-RBD response remains stable over time with a modest increase after the third dose. In line with this, a steady increase in the frequency of S-specific MBCs over three immunizations was observed, while the frequency of RBD-specific MBCs remained at post-dose 1 levels (Fig. 4H). Finally, we hypothesized that the relative decrease of RBD-specific IgG would be reflected in expanded responses towards other S domains. Indeed, anti-RBD IgG antibodies appeared rapidly after the first dose and remained at similar levels after each boost, while NTD- and S2-specific IgG significantly increased after the second dose. An additional increase in anti-S2, but not anti-NTD and -RBD antibodies, was detected after the third dose (Fig. 4I, J). Overall, this suggests that while the RBD-reactive B cell repertoire remained at consistently high levels throughout the study period, each boost immunization primarily expanded B cells of non-RBD specificity.

**Fig. 5:**
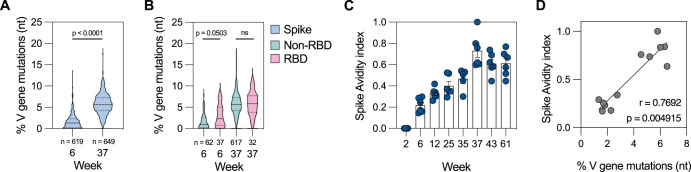
Maturation of the B cell response. **A** Somatic hypermutation in S-specific MBCs 2 weeks after boost 1 and 2. The number below represents the number of sequences included in the analysis at each timepoint. **B** Somatic hypermutation in Spike+ RBD+ and Spike+ RBD- MBCs 2 weeks after boost 1 and 2. The number below represents the number of sequences included in the analysis at each timepoint. **C** Avidity index of Spike-binding IgG plasma antibodies at different timepoints (*n* = 6), determined by chaotropic wash ELISA with 2 M NaSCN as the chaotropic reagent. **D** Correlation between average mutational load in circulating Spike-specific memory B cells and polyclonal serum antibody avidity (*n* = 6). Dashed line in the violin plots represents the median and dotted lines the quartiles (**A**, **B**). Data points are presented as mean ± SEM (**C**). Statistical analysis was performed using Kruskal-Wallis test (**A**), Kruskal-Wallis test with Dunn’s post hoc correction (**B**) or Spearman correlation (**D**).

Sequencing of the S-specific B cell receptor (BCR) repertoire after each boost showed a clear increase in somatic hypermutation (SHM) with the third dose. After dose 2, median SHM in S-specific BCRs was 1.4% (range 0.0–12.9%), which increased to 5.7% (range 0–17.7%) after dose 3 (Fig. 5A). Based on the single-cell sorting flow cytometry gates, we were able to differentiate RBD- and non-RBD-specific BCRs for a subset of sequenced MBCs. Whereas RBD-specific BCRs had higher SHM after dose 2 compared to non-RBD-specific BCRs, the mutational load was similar after dose 3 (Fig. 5B), which may be a result of the early expansion of RBD-specific MBCs after the first dose as demonstrated by our antibody and MBC probing data. Furthermore, avidity of S-binding circulating antibodies steadily increased over the course of 61 weeks (Fig. 5C) and correlated with the SHM load in S-specific MBCs after each immunization (Fig. 5D). The antibody avidity towards full S (Fig. 5C) and RBD monomers (Fig. 4E) increased at a similar rate, suggesting continued affinity maturation and a qualitative improvement despite the lack of a quantitative increase in anti-RBD antibodies.

### High degree of protection in immunized animals

**Fig. 6:**
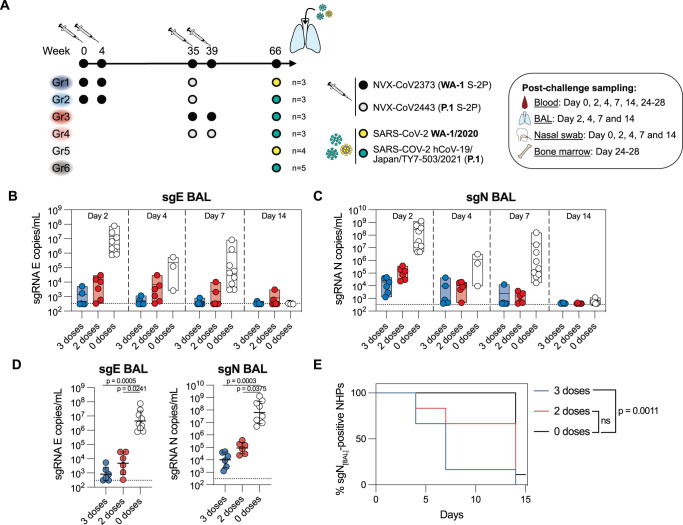
Durable protection induced by Novavax’s COVID-19 vaccine. **A** Study design and sampling schedule of the SARS-CoV-2 challenge experiment. Viral loads in BAL assessed by subgenomic (sg)E (**B**) and sgN gene-targeted RT-qPCR (**C**) (*n* = 6-9 per group). **D** Peak sgE and sgN loads in BAL during the 14 day follow-up after challenge (*n* = 6–9 per group). **E** Kaplan-Meier survival analysis, used to evaluate the time required for the animals in different groups to fully supress viral replication (sgN copies/mL < lower level of detection) (*n* = 6–9 per group). Data is presented as mean (min-max) (**B**, **C**) or geometric mean ± geometric SD (**D**). Statistical analysis was performed using Kruskal-Wallis test with Dunn’s post hoc correction (**D**) or log-rank Mantel-Cox test with Bonferroni correction. Dotted line corresponds to lower level of detection (**B**–**D**).

To evaluate long-term protection established by NVX-CoV2373 and NVX-CoV2443, rhesus macaques immunized zero, two or three times were challenged with a high dose (8 × 10^5^ PFU) of either USA-WA1/2020 or P.1 live virus ~7 months after the last immunization. Intranasal and intratracheal inoculation of the virus was performed as described previously in refs. ^27,53^. During the first 14 days after infection the animals were frequently sampled to assess both the viral loads and the elicited immune responses (Fig. 6A). Antibody titers in serum and BAL, as well as S-specific MBCs and Th1 cells were at comparable levels between subgroups at peak response and before challenge (Supplementary Fig. 5A–E). Post-challenge responses primarily differed based on the NHPs’ vaccination status, therefore, we grouped the animals based on the number of doses they received for the main analysis, regardless of vaccine or challenge virus strain.

Two days after challenge, immunized animals had ~3–4 log_10_ fewer copies of nucleocapsid (N) subgenomic RNA (sgRNA) in the lower respiratory tract compared to naïve animals (Fig. 6B, C). Peak sgN titers (the highest viral load detected during the 14-day follow-up) were significantly lower in immunized animals (Fig. 6D), and the animals that received three vaccine doses cleared the virus from the lower airways significantly faster than controls (Fig. 6E and Supplementary Fig. 6C). When divided by subgroup, the three-dose regimen performed well against both USA-WA1/2020 and P.1 (Supplementary Fig. 6A, B). Interestingly, there was a trend towards higher peak P.1 sgN copies in the animals that received two doses of P.1-based vaccine compared to WA-1-based vaccine (Supplementary Fig. 6A, B), however, the difference was not statistically significant due to the small sample size. The viral loads in the upper respiratory tract showed a similar pattern as that in the lower airways, with a significantly lower peak of sgN copies in the nasal cavity of immunized animals (Supplementary Fig. 6D–H).

**Fig. 7:**
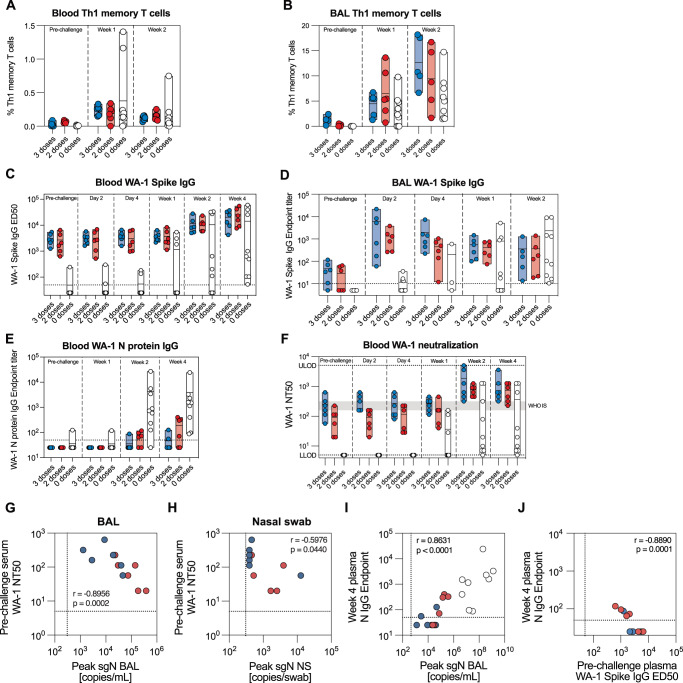
Rapid anamnestic responses in the lungs of immunized animals. Expansion of Spike-specific Th1 memory T cells in blood (**A**) and BAL (**B**) after challenge (*n* = 6–9 per group). WA-1 Spike-binding IgG antibodies in blood (**C**) and BAL (**D**) after challenge (*n* = 6–9 per group). **E** WA-1 N protein-binding IgG antibodies in blood after challenge (*n* = 6–9 per group). **F** Serum neutralization of WA-1/2020 SARS-CoV-2 virus after challenge (*n* = 6–9 per group). Gray shaded area represents the NT50 of WHO international standard NIBSC 20/136 (WHO IS). Correlation between pre-existing neutralizing serum antibodies and peak sgN viral load in the lungs (BAL) (**G**) and nasal cavity (nasal swab) (**H**) (*n* = 6–9 per group). **I** Correlation between peak viral load (sgN) in the lungs and the serum antibody response against N protein 4 weeks after challenge (*n* = 6–9 per group). **J** Correlation between pre-existing Spike-binding IgG and the serum antibody response against N protein 4 weeks after challenge (*n* = 6–9 per group). Data is presented as mean (min-max) (**A**–**F**). Dotted line corresponds to lower (**C**–**J**) and upper (**F**) level of detection. Statistical analysis was performed using Spearman correlation (**G**–**J**). LLOD lower level of detection, ULOD upper level of detection.

We sought to understand the immunological parameters driving the differences in the immune responses between groups. Th1 memory T cells, which also expanded in response to immunization, were elevated one and 2 weeks after challenge, especially in two naïve animals (Fig. 7A). Compared to values observed in the blood, all animals exhibited a greater increase in S-specific Th1 T cells in the BAL, with immunized animals displaying substantially higher frequencies than controls 2 weeks after challenge (Fig. 7B). In contrast, CD8 memory T cells were primarily induced in the naive group both in BAL and blood (Supplementary Fig. 7A, B), likely in response to a high degree of viral replication in the respiratory tract of these animals. S- and RBD-specific MBCs in the blood peaked 2 weeks after challenge, with the highest frequencies observed in the naïve group (Supplementary Fig. 7C, D). The IgG antibody titers in plasma followed a similar pattern, with the peak responses against S and RBD observed between weeks 2 and 4 after challenge (Fig. 7C and Supplementary Fig. 7E), whereas we observed a rapid expansion of S- and RBD-specific IgG antibodies in the lower airways of immunized animals as early as 2 days after challenge (Fig. 7D and Supplementary Fig. 7F). The IgA content in the BAL was too low to detect antigen-specific responses. However, analysis of S- and RBD-specific IgA in the blood showed low, but detectable titers at the time of challenge, which increased modestly 2 weeks after challenge (Supplementary Fig. 7G, H). Interestingly, 4 weeks after challenge we detected similar IgA responses between immunized and naïve NHPs (Supplementary Fig. 7G, H), whereas the IgG antibody levels were higher in immunized NHPs (Fig. 7C and Supplementary Fig. 7E). As NVX-CoV2373 and NVX-CoV2443 vaccines contain only the S protein, all animals were naïve to SARS-CoV-2 N protein before infection. While all naïve animals seroconverted against the N protein, only 2/6 and 4/6 NHPs in the three and two dose groups, respectively, developed detectable anti-N antibodies (Fig. 7E). Moreover, the overall anti-N protein antibody titers were higher in the control group compared to vaccinated groups.

Finally, mean neutralizing antibody titers in the serum of the immunized NHPs 7 months after the last immunization persisted at the levels equivalent to the WHO International standard, with 2.8-fold higher titers in the 3-dose group than in the 2-dose group (mean NT_50_ = 252.7 vs 91.5, respectively, Fig. 7F). Two weeks after challenge, neutralizing antibody titers in all immunized animals exceeded the WHO standard, however there was a large spread in the naïve group. Rapid control of viral infection in immunized animals is likely a result of the rapid anamnestic response in the lungs, evident by substantial antibody and T cell responses in the lower airways as early as 2 days after challenge, combined with a potent pre-existing immunity in the form of circulating antibodies. Indeed, pre-challenge neutralizing titers were negatively correlated with peak viral loads in lower (BAL) and upper (NS) respiratory tract (*r* = −0.8956, *p* = 0.0002 and *r* = −0.5976, *p* = 0.0440, respectively) (Fig. 7G, H). On the other hand, seroconversion against the N protein was positively correlated to the peak viral titers in BAL (Fig. 7I) and negatively correlated to the pre-existing S-binding antibodies (Fig. 7J), suggesting that in the immunized NHPs, the N protein antigen load was not sufficient to elicit a detectable immune response after the rapid suppression of viral replication. Altogether, this provides evidence for a durable protection induced by NVX-CoV2373 and NVX-CoV2443 COVID-19 vaccines.

## Discussion

Large-scale clinical trials are the ultimate test of vaccine efficacy but provide limited information on the immune mechanisms driving the efficacious responses. Here, we utilized the non-human primate model to perform an in-depth study of innate and adaptive immune responses to Matrix-M^TM^ adjuvanted Novavax’s COVID-19 vaccine, with long-term sampling including BAL for mucosal responses. Although Matrix-M^TM^ and other saponin-based adjuvants have proven to be highly immunogenic in NHPs^12,18,20,26,54–56^ and humans^16,19,57^, early innate events remain poorly understood. A recent successful phase IIb trial of Matrix-M^TM^-containing malaria vaccine R21^58^ highlights the need for mechanistic understanding of this adjuvant.

Systemic type I interferon responses induced by mRNA vaccines have been implicated in promoting longevity of immune responses to vaccination^59–61^. In small animal models, saponin-containing adjuvants have been shown to primarily promote trafficking of immune cells into the dLNs and activation of local APCs, with limited transcriptional changes and low levels of pro-inflammatory cytokines detected in the periphery^20–22,55,62,63^. At the site of injection, Matrix-M^TM^ promotes infiltration of innate immune cells compared to PBS^12,64^, supported by our RNA sequencing data. In NHPs, major transcriptomic changes were detected only at the injection site and in dLNs, but not in the blood, corroborating previous findings that after Matrix-M^TM^ immunization, inflammation is limited primarily to the local vaccine-draining sites. Clinical studies focusing on Matrix-M^TM^-induced innate responses are needed to fully address the mechanisms behind reported adverse effects.

Characterization of B and T cell responses showed similar immune profiles elicited by mRNA and Novavax’s vaccine 4–6 months after the first immunization^41^. While several studies have addressed the RBD/non-RBD dichotomy in B cell repertoires after completing an mRNA immunization series^30,41,43^, our analysis comprehensively profiled B cell responses after each protein subunit immunization including early and late timepoints. After two immunizations in humans, RBD-specific MBCs make up 20–40% of the total S-specific MBC pool^30,41,43,65^, consistent with our observations at week 6, where 36.4% (range 26.9–48.6%) of all S-specific MBCs bound RBD. However, after the first immunization in NHPs, we found that the majority of S-specific MBCs were also RBD-specific, in contrast to previous reports in human cohorts where the proportion of RBD-specific MBCs was relatively stable across immunizations^30,43,66^. Since pre-existing immunity to beta coronaviruses likely shapes the B cell repertoire after COVID-19 immunization or infection by boosting responses to the conserved S2 domain^44,67–69^, this should be investigated in a cohort of Novavax’s vaccinees.

Through mapping of the serum response to S subdomains, we showed marked immunodominance of RBD-directed antibodies after the first immunization. RBD-specific antibodies confer the majority of virus neutralization^32,33^, likely contributing to rapid development of potent neutralizing responses 2 weeks after a single NVX-CoV2373 immunization. The relative decrease in proportion of RBD-specific IgG antibodies and MBCs coincided with expansion of NTD- and S2-specific antibodies after the second and third dose. High titers of pre-existing RBD-specific antibodies may limit activation and expansion of B cells with the same specificity through negative feedback mechanisms^47,48,70,71^ and drive the expansion of non-RBD-specific B cells. This finding has possible implications for the development of RBD-based COVID-19 booster vaccines. Our data suggests that expansion of non-RBD-specific B cells does not hinder development of broad neutralization potency against Omicron subvariants, while elicitation of non-neutralizing antibodies against the conserved epitopes of the S protein can contribute to mitigating infection or severe disease through Fc receptor functions^44,72,73^, particularly in the face of rapid viral evolution.

Accumulation of SHM, combined with evidence of persistent germinal centers after mRNA^74^ and protein immunization^75^, can greatly contribute to affinity maturation of vaccine-specific responses even in the absence of a booster dose^76^. In our study, maturation of the RBD-specific B cells was apparent through an increase in serum antibody avidity and mutational load in RBD-specific MBCs in the absence of a quantitative increase. Similar observations were made in mRNA-vaccinated human cohorts^76–78^. While the third immunization further increased SHM and antibody avidity, major affinity maturation can be attributed to the first booster dose as evident by the progressive increase in S antibody avidity between weeks 6 and 35. Importantly, the second boost elevated affinity-matured serum antibodies through the plasmablast response and expanded the long-lived plasma cell population in the bone marrow, substantially contributing to long-lived protective immunity.

Increased cross-reactivity, avidity and potency of serum antibodies after the third immunization highlight the importance of the booster for eliciting high-quality immune responses. It has been suggested that three exposures to the S protein, through immunization or infection, are required for generation of high-affinity serum antibodies and enhanced neutralization breadth^79–82^, likely due to the repetitive rounds of affinity maturation in the germinal centers. Moreover, clinical studies of Matrix-M^TM^-adjuvanted vaccines hypothesize epitope spreading as one of the mechanisms contributing to enhanced neutralization breadth^83,84^. While the third, heterologous immunization in our study qualitatively and quantitatively improved the antibody response against S, and in particular cross-neutralization of P.1, BA.2, and BA.5 strains, we did not have a comparator group receiving the homologous booster due to the limited number of available NHPs. A few clinical studies reported minor benefits of using bivalent boosters^85,86^, but the majority of pre-clinical and clinical studies found no significant differences in the elicited immune responses of subjects receiving a single ancestral or variant-based COVID-19 booster^87–91^. This suggests that protein subunit vaccines as a platform can elicit high-quality antibody responses with neutralization of forward drift variants as evident by potent neutralization of BA.2 and BA.5 Omicron strains after the third dose, with one of the lowest fold-changes compared to WA-1 reported to date^76,77,92,93^.

Exposure to infectious virus at peak immunity after immunization is rare. To simulate the viral exposure in the general population, the animals in our study were challenged 7 months after the last vaccine dose when antibody titers waned to maintenance levels. Even though none of vaccinated NHPs were fully protected from infection, there was a striking difference in peak viral titers of 3-4 log_10_ between immunized and naïve animals. A year after two mRNA-1273 immunizations, Delta challenged NHPs showed similarly high viral titers 2 days after challenge as controls, followed by an expedited viral clearance during days 4–7 attributed to local re-activation of virus-specific memory B cells^27^. Higher viral loads at an early timepoint may be due to higher infectivity of the Delta variant^94^, the two-dose vaccine schedule as well as a longer period between immunization and challenge compared to our study. Although it has been shown that the B cells undergo affinity maturation for weeks to months after boost immunizations with mRNA or protein vaccines^71,74–76^, the third dose appears to be critical for enhancing antibody avidity and VOC cross-neutralization.

The correlate of protection for SARS-CoV-2 infection has yet to be established, however several studies, including ours, show strong correlation between pre-existing binding and/or neutralizing antibody titers and viral loads in the respiratory tract after infection^53,95–97^. S-specific MBCs can rapidly differentiate into antibody-secreting cells upon antigen re-exposure and thus their persistence is critical for efficient memory responses. In our study, S-specific MBCs after NVX-CoV2373/2443 immunization persisted at low, but stable levels until challenge. Pre-existing S-specific memory B cells have been shown to correlate with post-vaccination antibody response^30^, highlighting that stable populations of vaccine-elicited S-specific MBCs are needed for swift antibody responses to infection or vaccination.

While the prevention of infection primarily relies on the neutralizing antibody responses, CD8 T cells have been shown to contribute to virologic control and limit viral replication^95,97–99^. NHPs in this study developed strong S-specific CD4 and CD8 T cells responses in the lungs after challenge, in addition to the anamnestic antibody responses, indicating that a concerted memory response can lead to rapid control of viral replication several months after last immunization.

Protection from infection likely requires both a sufficient antibody titer and potency for rapid viral control. Saponin-based adjuvants were shown to induce superior durability of serum antibodies^55^, which may offer superior long-term protection. In a study that directly compared mRNA and protein subunit COVID-19 vaccines in NHPs, two doses of squalene-based AS03-adjuvanted subunit vaccine-elicited more durable neutralizing titers over 6 months, although the vaccine dosage was inconsistent with clinical doses^100^. Taken together, these data suggest protein vaccines, eliciting durable antibody titers, may perform better than mRNA vaccines as yearly SARS-CoV-2 boosters.

Collectively, we showed that Novavax’s Matrix-M^TM^-adjuvanted protein vaccines induce limited systemic innate events, both in terms of cytokine production and transcriptomic changes. NVX-CoV2373/2443 immunization elicited highly potent and durable antibody responses, with enhanced breadth after each dose. Seven months after immunization, significant protection from high dose viral challenge was observed in vaccinated NHPs, mediated by a rapid anamnestic response in the mucosal tissues. Novavax’s COVID-19 protein vaccine can therefore effectively serve as a primary or booster dose for eliciting high-quality vaccine immunity.

### Limitations

The small sample size of groups in our study, especially in the challenge phase, was not sufficient to discern the efficacy of each immunization regimen individually. The scarce supply of NHPs as a result of the COVID-19 pandemic did not allow us to compare all combinations of homologous and heterologous immunization strategies, requiring us to limit the study design to the most relevant vaccine combinations and study timelines at the time. Nevertheless, our study resembles the immunization schedules administered in the clinical practice and highlights the importance of vaccine-induced immunity in controlling and clearing SARS-CoV-2 infection.

## Methods

### Vaccine production

Full-length S glycoprotein gene sequence (GenBank MN908947 nucleotides 21563–25384) for NVX-CoV2373 was synthetically produced and codon optimized for expression in *Spodoptera frugiperda* (Sf9) cells (GenScript) as previously described in ref. ^18^. Briefly, the S1/S2 furin cleavage site 682-RRAR-685 was modified to 682-QQAQ-685 and two proline substitutions were introduced at positions K986P and V987P (2P) to stabilize the full-length SARS-CoV-2 S^101^. For NVX-CoV2443, the following substitutions were introduced in addition to the stabilizing 3Q-2P mutations: L18F, T20N, P26S, D138Y, R190S, K417T, E484K, N501Y, D614G, H655Y, T1027I, V1176F. Matrix-M^TM^ was supplied by Novavax AB (Uppsala, Sweden). Recombinant protein nanoparticles, Matrix-M^TM^ and formulation buffer (25 mM sodium phosphate, 300 mM sodium chloride, 0.01% Tween 80, pH 7.2) were mixed immediately before administration.

### Rhesus macaque model

Animal experiments were conducted following the guidelines and regulations of the Association for Assessment and Accreditation of Laboratory Animal Care, the Swedish Animal Welfare Agency and the European guidelines for animal care. The study was approved by the regional animal ethics committee of Northern Stockholm, institutional ethical committee “Comité d’Ethique en Expérimentation Animale du Commissariat à l’Energie Atomique et aux Energies Alternatives” and the French Ministry of Higher Education and Research. Indian rhesus macaques (*Macaca mulatta*, male and female, 4–6 years old at study start) used in immunization experiments (*n* = 12) and as control animals (*n* = 3) were housed at Astrid Fagraeus Laboratory at Karolinska Institutet (Stockholm, Sweden). Six naïve Chinese rhesus macaques (*Macaca mulatta*, male, 3–4 years old at study start), serving as additional control animals, were housed at the IDMIT animal facility (CEA, Fontenay-aux-Roses, France). The animals received intramuscular injections according to the study schedule in the left quadriceps, consisting of 5 μg protein nanoparticles (NVX-CoV2373 or NVX-CoV2443) and 50 μg Matrix-M^TM^. At week 66, all animals were challenged with a total dose of 8 × 10^5^ PFU SARS-CoV-2 (isolate USA-WA1/2020 (BEI: NR-53872) or hCoV-19/Japan/TY7-503/2021 (BEI: NR-55364)). The inoculum was diluted in PBS and administered 3 mL intratracheally and 1 mL intranasally (0.5 mL in each nostril) as described previously in refs. ^27,53^. Heparinized peripheral blood, serum, bone marrow aspirates and bronchoalveolar lavage (BAL) were collected as depicted in Fig. 2A and Fig. 6A. Body weight and temperature were monitored at each sampling timepoint. Six of the control animals, housed at CEA, were not sampled for BAL at day 4 post challenge due to restrictions in the ethical permit.

Two female Chinese rhesus macaques (*Macaca mulatta*, 4–5 years old) were immunized with 75 μg Matrix-M^TM^ or PBS as a part of another study^12^. To maximize sample collection, each animal received an immunization in each limb muscle (deltoid or quadriceps) and PBS injections in calves. Tissue biopsies of injection sites and dLNs were collected 24 h after immunization and stored in RNALater (Invitrogen) at −20 °C until use.

### Safety measurements

Complete blood counts and clinical chemistry analyses were performed at baseline, 24 h and 14 days after the first immunization by Adlego Biomedical (Solna, Sweden). Clinical chemistry was performed using an Abaxis Vetscan VS2 3.1.35 chemistry analyzer with mammalian liver profile rotors (Triolab).

### Sample processing

A standard gradient density centrifugation using Ficoll-Paque (GE Healthcare) was used to isolate peripheral blood mononuclear cells (PBMCs) from heparinized blood. PBMCs were either cryopreserved in 10% dimethyl sulfoxide (DMSO)/fetal calf serum (FCS) or immediately used for downstream applications. Heparinized bone marrow samples were processed in the same way as blood, and additionally passed through a 70 μm cell strainer before use. BAL cells were separated from the supernatant by centrifugation and filtration through a 70 μm cell strainer, then were used fresh in a T cell recall assay. BAL fluid was concentrated 10-fold using Amicon Ultra centrifugal filter units with a 30 kDa cutoff (Millipore) before downstream analysis.

### Innate immunoprofiling

On days 0, 1, and 14 after the first immunization, the cellular composition of the PBMCs was analyzed using flow cytometry. Freshly isolated PBMCs were stained with Live/Dead Fixable Blue Dye (Life Technologies, cat# L-23105, 1:40 dilution) and FcR blocking reagent (Miltenyi Biotec, cat# 130-059-901, 1:20 dilution) followed by a panel of antibodies: CD40 FITC (5C3, Biolegend, cat# 334306, 1:20 dilution), NKG2A PE (Z199, Beckman Coulter, cat# IM3291U, 1:40 dilution), CD80 BV421 (L307.4, BD, cat# 564160, 1:40 dilution), CCR7 PE-Dazzle594 (G043H7, Biolegend, cat# 353236, 1:50 dilution), CD123 Per-CP-Cy5.5 (7G3, BD, cat# 558714, 1:80 dilution), CD3 APC-Cy7 (SP34-2, BD, cat# 557757, 1:80 dilution), CD66 APC (TET2, Miltenyi Biotec, cat# 130-118-539, 1:80 dilution), CD70 BV786 (Ki-24, BD, cat# 565338, 1:80 dilution), HLA-DR BV650 (L243, Biolegend, cat# 307650, 1:80 dilution), CD11c PE-Cy7 (3.9, Biolegend, cat# 301608, 1:160 dilution), CD16 AF700 (38 G, BD, cat# 560713, 1:160 dilution), CD20 BV605 (2H7, Biolegend, cat# 302334, 1:160 dilution) and CD14 BV510 (M5E2, Biolegend, cat# 301842, 1:160 dilution). After washing with PBS, samples were fixed using 1% paraformaldehyde (PFA) and acquired on a BD LSRFortessa cell analyzer. The data were analyzed using FlowJo software v.10.7.1 (FlowJo).

### Plasma cytokine and chemokine quantification

After the first immunization, plasma cytokines and chemokines were analyzed using the ProcartaPlex NHP Cytokine & Chemokine Panel 30plex (Thermo Fisher Scientific) according to the manufacturer’s instructions at the Affinity Proteomics core facility, SciLifeLab, Stockholm, Sweden. Samples collected at baseline, 24 h, and 14 days after the immunization were analyzed using a MagPix (Luminex) instrument, and the data were analyzed with Belysa Immunoassay Curve Fitting software (Millipore). Standard curves were generated using 5-parameter logistic curve fit.

### RNA sequencing and bioinformatic analysis

At 0 and 24 h after the first immunization, whole blood was collected into PAXgene Blood RNA tubes (PreAnalytiX) and stored at −20 °C. Tissue biopsies, collected 24 h after 75 μg Matrix-M^TM^ or PBS immunization, were placed in RNALater (Invitrogen) and stored at −20 °C. RNA isolation was performed using PAXgene Blood RNA Kit (PreAnalytiX) for blood samples and RNeasy mini kit (Qiagen) for lymph nodes according to manufacturer´s instructions. For muscle biopsies, RNA was isolated using RNeasy Fibrous Tissue mini kit (Qiagen) according to the manufacturer’s instructions. RNA integrity was checked using TapeStation RNA ScreenTape assay (Agilent Technologies) according to the manufacturer’s instructions. In preparation for Illumina sequencing, isolation of mRNA, cDNA synthesis, anchor ligation, amplification and library indexing were performed using the Illumina Stranded mRNA Prep Ligation kit according to the manufacturer’s instructions. The libraries were sequenced using an Illumina NovaSeq S6000 on one lane of the S4-300 (v1.5) flowcell with the standard paired-end read set up (2 × 150 bp), with an average sequencing depth of 38 M reads per sample.

Samples were preprocessed using an nf-core rnaseq pipeline (v3.7)^102^. STAR alignment (v2.7.10a) to the *M. mulatta* genome (Mmul_10) was used for genome alignment, and quantification was performed with Salmon (v1.8.0). A customized bioinformatic analysis workflow in R (v4.1.2) was used: differential gene expression analysis was performed using DESeq2 (v1.34.0), ClusterProfiler (v4.2.2) was used for Gene Set Enrichment analysis in combination with the Blood Transcriptome Module database^103^. To compare differentially expressed genes, a Wald test was performed with multiple hypothesis testing controlling the false discovery rate using the Benjamini-Hochberg procedure (*q* < 0.05).

### Spike detection in plasma by MSD

An S-PLEX SARS-CoV-2 Spike Kit (K150ADJS, Meso Scale Diagnostics) was used to quantify the Spike concentration in plasma before and after immunization according to the manufacturer’s instructions at the Affinity Proteomics core facility, SciLifeLab, Uppsala, Sweden. 25 μL of plasma was used for the analysis, and the plates were read using a MESO QuickPlex SQ 120 instrument. Quantification was based on an 8-point calibration standard curve using the recombinant SARS-CoV-2 S protein included in the kit. The raw signals were converted into data expressed in femtograms per milliliter.

### Binding antibody titers by ELISA

96-well half-area ELISA plates (Greiner Bio) were coated with recombinant proteins (prefusion-stabilized S (S-2P) or RBD, acquired through the Global Health Discovery Collaboratory funded by the Bill & Melinda Gates Foundation) at 1 μg/mL in PBS and incubated overnight at 4 °C. Plates were washed three times using PBS-T (PBS containing 0.05% Tween 20) and blocked with blocking buffer (PBS with 5% (w/v) skimmed milk powder) for 1 h at room temperature (RT). Duplicates of serially diluted samples in blocking buffer were added to the plate and incubated for 2 h at RT. The plates were washed three times and goat anti-monkey IgG-horseradish peroxidase (Nordic MUBio, cat# 246-GAMon/IgG(H + L), 1:20 000 dilution) in blocking buffer was added for 1 h at RT. For development, 1-Step Ultra TMB-ELISA substrate (Thermo Fisher Scientific) was added for 5 min and the reaction was stopped with 1 M H_2_SO_4_. The absorbance was measured at 450 nm with background correction at 570 nm. Data was analyzed with Prism v9.4.1 using 4-parameter logistic curve fit.

### Competition ELISAs

RBD and P.1 Spike competition ELISAs were performed as above with some modifications. For the P.1 competition ELISA, the plates were coated using BV2373 protein (WA-1 Spike, Novavax). Serially diluted plasma samples were pre-incubated with 20 μg/mL of competitor protein (RBD or BV2443 (P.1 Spike)) or blocking buffer for 30 min, and then transferred to the ELISA plates for further 1.5 h incubation. The proportion of competition was calculated based on the decrease in ED_50_ value between the condition with and without the competitor.

For the competition assay using characterized monoclonal antibodies (mAbs), the antibody sequences were retrieved from the literature^104–108^ and recombinant antibodies were produced by GenScript (Leiden, Netherlands) or Institute for Protein Design (Seattle, WA, USA). The antibodies were biotinylated using EZ-Link Micro Sulfo-NHS-LC Biotinylation Kit (Thermo Fisher Scientific) according to the manufacturer’s instructions.

The ELISA was performed as above, with some modifications. The experimental conditions were optimized for each competition mAb separately to maximize the dynamic range. ELISA plates were coated with 1 μg/mL S-2P for B38, C144, S2M11 and S309 and 2 μg/mL S-2P for CR3022. Serial dilutions of plasma in duplicates were added to the ELISA plates for 30 min to allow for binding of plasma antibodies, followed by an equal volume of biotinylated competitor antibodies at a predetermined concentration in blocking buffer for further 1.5 h incubation at RT. The detection was performed using Pierce high sensitivity NeutrAvidin-horseradish peroxidase (Thermo Fisher) at a 1:5000 dilution in PBS, and plates were developed using 1-Step Ultra TMB-ELISA substrate (Thermo Fisher). Competition between unbiotinylated and biotinylated competitor antibodies served as standard curve. Levels of competitor-like antibodies in the plasma were calculated by multiplying the ED_50_ of unbiotinylated mAbs with the ED_50_ of each sample.

### Antibody avidity assay

An avidity ELISA was performed as above, with some modifications. After sample incubation and washing with PBS-T, the samples were treated with 2 M NaSCN (Sigma-Aldrich) or PBS for 10 min. Detection and development were performed as described. The avidity index was calculated as a ratio between ED_50_ values of NaSCN- and PBS-treated conditions. If ED_50_(NaSCN) was below the level of detection, an avidity index of 0 was assigned. On the contrary, if ED_50_(NaSCN) > ED_50_(PBS), an avidity index of 1 was assigned.

### Neutralization and pseudovirus neutralization assays

Live virus neutralization assay using an authentic wild type SARS-CoV-2 virus (strain 2019-nCoV strain 2019-nCov/Italy-INMI1. European Virus Archive Global (EVAg), Marseille, France) was performed at VisMederi srl (Siena, Italy) as previously described in ref. ^109^, and pseudovirus particle neutralization assay using vesicular stomatitis virus (VSV)ΔG S pseudotyped virus with a luciferase reporter was performed at Nexelis (Laval, Canada) as described previously in ref. ^110^. All samples were assayed in duplicates.

Neutralization of variants of concern was assessed as previously described in ref. ^111^. Briefly, WA-1, P.1, BA.2, and BA.5 Spike-pseudotyped lentivirus particles delivering a luciferase reporter (standardized to ±100 000 RLU) were pre-incubated with three-fold serial serum dilutions for 1 h at 37°C in a black-walled 96-well plate in duplicates. 10 000 HEK293T-ACE2 cells were added to each well and the plates were incubated at 37°C and 5% CO_2_ for 48 h. Luminescence was measured using Bright-Glo substrate (Promega) using GloMax Navigator Luminometer (Promega). Effective 50% neutralization titer was calculated based on the luminescence of infected control wells in the absence of serum.

### Systems serology

Antigen-specific antibody subclass isotypes and FcγR binding were analyzed by Luminex multiplexing in plasma samples collected at weeks 0, 2, 6, 37 and 61 from the 3-dose group. Antigens (BV2373, BV2443 (Novavax), WA-1 Spike, RBD, NTD and S2, B.1.351 Spike and RBD, B.1.617.2 Spike and RBD, B.1.1.529 Spike and RBD, HKU Spike, 229E Spike, MERS Spike (all Sino Biological)) were coupled to magnetic Luminex beads by carbodiimide-NHS ester coupling with an individual region per antigen according to manufacturer’s instructions. Antigen-coupled beads were incubated with different plasma dilutions to form immune complexes. Mouse anti-rhesus detection antibodies were added for each Ab isotype (total IgG (Southern Biotech), total IgM (Life Diagnostic), IgG1, IgG2, IgG3, IgG4, total IgA (all NIH Nonhuman Primate Reagent Resource)), followed by PE-conjugated anti-mouse Fc IgG antibody (Thermo Fisher). FcR-binding was quantified in a similar way by coupling PE-streptavidin (Agilent Technologies) to recombinant and biotinylated NHP FcRs (FcγR2A-1, FcγR2A-2, FcγR2A-3, FcγR2A-4, FcγR3A, courtesy of Duke Protein Production Facility) which were used as secondary probes. Relative antibody concentration per antigen was determined on an iQue Screener (IntelliCyt), and analysis was performed on IntelliCyt ForeCyt (v 8.1).

Bead-based assays were used to quantify antibody (Ab) functionality. Ab-dependent cellular phagocytosis (ADCP)^112^, Ab-dependent neutrophil phagocytosis (ADNP)^113^, and Ab-dependent complement deposition (ADCD)^114^ were measured as described previously. BV2373, BV2443 (Novavax), SARS-CoV-2 WA-1, and B.1.1.529 Omicron S protein (Sino Biological) were coupled to yellow-green fluorescent (ACDP, ADNP) or non-fluorescent (ADCD) neutravidin beads (Invitrogen) and incubated with plasma samples to form immune complexes. For ADCP, cultured human monocytes (THP-1 cell line) were incubated with immune complexes for 18 h at 37 °C, during which phagocytosis occurred. For ADNP, neutrophils were isolated from fresh whole blood using EasySep Direct Human Neutrophil Isolation kit (StemCell) and incubated with immune complexes for 30 min at 37 °C. Neutrophils were stained with an anti-CD66b Pacific Blue antibody (Biolegend) prior to flow cytometry. For ADCD, lyophilized guinea pig complement (Cedarlane) was reconstituted according to the manufacturer’s instructions, diluted in a gelatin veronal buffer (Boston BioProducts) and added to the immune complexes for 50 min at 37 °C. C3 that bound to immune complexes was detected with a FITC-conjugated goat anti-guinea pig complement C3 (MP Biomedicals) antibody. Flow cytometry acquisition of all assays was performed using a Stratedigm 1300EXi cytometer. For ADCP and ADNP, phagocytosis events were gated on bead-positive cells, with additional pre-gating step for CD66b+ cells for ADNP. A phagocytosis score for ADCP and ADNP was calculated as (percentage of FITC+ cells) * (the geometric mean fluorescent intensity (gMFI) of the FITC+ cells)/10,000. ADCD was reported as gMFI of FITC-anti-C3.

Principal component analysis (PCA) was constructed with FactoMineR R package v2.7^115^ using all collected antibody features as variables. The co-correlate network analysis was performed using a systemsseRology package (https://github.com/LoosC/systemsseRology)^45^. Features were selected using the least absolute shrinkage and selection operator (LASSO) algorithm. All antibody features were used as input and the LASSO selection was repeated 100 times. Only features that were selected in 80% of the trials were used in the co-correlate network analysis. The co-correlate network was built using a threshold of absolute Spearman rho greater than 0.7 and BH-adjusted p-value lower than 0.05.

### Vaccine-specific memory B cell quantification and isolation

Recombinant S-2P, RBD and BV2443 proteins were biotinylated using EZ-Link Micro Sulfo-NHS-LC Biotinylation Kit (Thermo Fisher Scientific) according to the manufacturer’s instructions. Biotinylated proteins were coupled to streptavidin-conjugated fluorophores (SA-PE, SA-APC or SA-BV421) to generate molecular probes. PBMCs were stained with 100 ng fluorescent protein probes for 20 min at 4°C, followed by 7-aminoactinomycin D (7-AAD, Thermo Fisher, cat# A1310, 1:4000 dilution) and a panel of antibodies: IgM PerCP-Cy5.5 (G20-127, BD, cat# 561285, 1:40 dilution), CD3 BV510 (SP34-2, BD, cat# 740187, 1:40 dilution), CD123 BV510 (6H6, Biolegend, cat# 306022, 1:40 dilution), CD16 BV510 (3G8, BD, cat# 563830, 1:80 dilution), HLA-DR BV650 (L243, Biolegend, cat# 307650, 1:80 dilution), IgG BV786 (G18-145, BD, cat# 564230, 1:80 dilution), CD20 BV605 (2H7, Biolegend, cat# 302334, 1:160 dilution), CD14 BV510 (M5E2, Biolegend, cat# 301842, 1:60 dilution) and IgD FITC (polyclonal, Southern Biotech, cat# 2030-02, 1:160 dilution), for another 20 min at 4°C. Cells were washed with PBS with 2% heat-inactivated FCS and fixed with 1% PFA. Samples were acquired on a BD LSRFortessa cell analyzer and the data were analyzed using FlowJo software v.10.7.1 (FlowJo).

At peak timepoints (week 6 and 37), antigen-specific IgG+ B cells were single-cell sorted into 96-well plates for isolation of vaccine-specific BCRs. The procedure was similar as above, except that the PBMCs were not fixed after staining and instead resuspended in complete medium (RPMI 1640 medium with 10% heat-inactivated FCS, 100 U/mL penicillin, 100 mg/mL streptomycin, and 2 mM L-glutamine) with 7-AAD. BD Aria III Fusion cell sorter was used for single-cell sorting of Lin^-^ HLA-DR^+^ CD20^+^ IgM^-^ IgG^+^ B cells, double positive for S protein. The plates were immediately frozen on dry ice for subsequent BCR amplification. At week 6, the samples were probed using S-2P PE, S-2P APC and RBD BV421. At week 37, BV2443 PE and BV243 APC were added to the probe mix. Index sorting data is available for selected plates, to determine RBD specificity of sorted cells.

A subset of Spike-specific BCR sequences at week 6 was acquired using FACS sorting combined with 10x Genomics single-cell RNA sequencing. Each sample was labeled using TotalSeq-C anti-human hashing antibodies (Biolegend) prior to cell sorting. Dual-S-positive IgM- B cells were sorted and processed with the Chromium Single Cell V(D)J Enrichment Kit, Human B Cell (10x Genomics), sequencing ‘5’ V(D)J’ enriched libraries and TotalSeq-C feature barcode libraries. Rhesus macaques are not directly supported by the 10x kits, so a mix of primers targeting the Ig constant region^116^ was spiked in during the two enrichment PCR steps (Supplementary Table 1). 5 μL of each 100 μM primer was added to MasterMix1 and MasterMix2 for enrichment steps 1 and 2, respectively, and diluted to a final volume of 50 μL. 5 μL of each MasterMix was added to the PCR mixture replacing 5 μl of nuclease-free water. 10X libraries were sequenced on a NovaSeq6000 using NovaSeq XP workflow in SP mode flowcell.

### Isolation of Spike-specific B cell receptors and B cell repertoire analysis

BCR sequences were recovered from single-cell sorted B cell with reverse transcription using random hexamers (Invitrogen) as previously described in ref. ^51^. Superscript III reverse transcriptase kit (Invitogen) was used according to manufacturer’s instructions. Nested PCR protocol for amplification of heavy and light chain transcripts was performed as reported previously in ref. ^117^. PCR products were Sanger sequenced by Genewiz (Leipzig, Germany). The resulting chromatograms were pre-processed with scifer package (v0.99.3)^118^ and the high-quality sequences were aligned to the KIMDB rhesus database (v1.0)^119^ using IgDiscover (v0.15.1)^120^.

Raw sequencing reads from the 10x libraries were processed with CellRanger (v3.1.0, 10x Genomics). Demultiplexing was performed based on cell hashing with barcoded antibodies using Seurat R package (v4.0.6). We removed cells with less than 200 detected genes, mitochondrial reads higher than 20%, ribosomal reads lower than 5%, and all genes needed to be expressed in at least three cells. Doublets were detected and removed using DoubletFinder R package (v2.0.3). VDJ contigs were assembled using CellRanger’s *--denovo* option, and assembled contigs were subsequently re-aligned using IgDiscover (v0.13.0) and filtered based on default settings. Sequences were aligned to the KIMDB rhesus macaque database^119^. In total, 182 paired heavy and light chains were retrieved.

### Detection of antigen-specific antibody-secreting cells

Antigen-specific antibody-secreting cells in the bone marrow and peripheral blood were assessed by ELISpot at indicated timepoints as previously described in ref. ^51^. Multiscreen IP filter plates ELISpot 96-well plates (Millipore) were activated using 35% ethanol, washed with PBS and coated with Affinity Pure goat anti-human IgG Fc fragment-specific capture antibody (Jackson ImmunoResearch, cat# 109-005-008) at 1 μg/mL in PBS. After an overnight incubation at 4°C, plates were washed with PBS and blocked using complete medium. Freshly isolated PBMCs or bone marrow mononuclear cells were plated in serial dilution and incubated overnight at 37°C and 5% CO_2_. After incubation, plates were washed with PBS-T and biotinylated protein probes were added for 1.5 h (0.25 μg/mL goat anti-human IgG Fc fragment-specific antibody (Jackson ImmunoResearch, cat# 109-065-008), 1 μg/mL S-2P/RBD/ovalbumin (OVA)). After another wash with PBS-T, streptavidin-conjugated alkaline phosphatase (Mabtech) was added at 1:1000 dilution for 30 min. The plates were developed using nitro blue tetrazolium 5-bromo-4-chloro-3’ indolyphosphate substrate (Mabtech) for 7 min. The spots were counted using an AID ELISpot reader (Autoimmun Diagnostika) and background subtraction was performed based on OVA wells.

### Memory T cell recall assay

Frequencies of S-specific memory T cells in blood and BAL were assessed using a re-stimulation assay as described previously using 2 μg/mL PepMix SARS-CoV-2 Spike overlapping peptides in DMSO (15mers with 11 amino acid overlap, JPT Peptide Technologies)^51^. The antibody panel used for surface staining was: CD103 FITC (2G5, Beckman Coulter, cat# B49222, 1:50 dilution), CCR7 BV421 (G043H7, Biolegend, cat# 353208, 1:50 dilution), CD8a BV711 (RPA-T8, Biolegend, cat# 301044, 1:80 dilution), CD4 PE-Cy5.5 (S3.5, Invitrogen, cat# MHCD0418, 1:80 dilution) and CD45RA BV650 (5H9, BD, cat# 740608, 1:500 dilution), and the intracellular proteins were stained using: IL-21 AF647 (3A3-N2.1, BD, cat# 560493, 1:20 dilution), IL-13 PE (JES10-5A2, BD, cat# 559328, 1:33 dilution), IL-2 BV605 (MQ1-17H12, BD, cat# 564165, 1:50 dilution), IL-17A BV785 (BL168, Biolegend, cat# 512338, 1:67 dilution), CD69 ECD (TP.1.55.3, Beckman Coulter, cat# 6607110, 1:67 dilution), CD3 APC-Cy7 (SP34-2, BD, cat# 557757, 1:200 dilution) and IFNγ AF700 (B27, Biolegend, cat# 506516, 1:200 dilution). Acquisition was performed using BD LSRFortessa cell analyzer, and the data were analyzed using FlowJo software v.10.7.1 (FlowJo).

### Quantification of subgenomic RNA after challenge

Viral loads in the respiratory tract, evaluated as a copies of subgenomic (sg)E and sgN RNA in the sample, were measured as previously reported^27,53^. BAL fluid and nasal swabs were stored in RNAzol BD (Molecular Research Center) and PBS until use, respectively. Total RNA was extracted with the RNAzol BD column kit (Molecular Research Center). TaqMan Fast Virus 1-Step Master Mix (Applied Biosystems), gene-specific primers (sgLeadSARSCoV2_F: 5′-CGATCTCTTGTAGATCTGTTCTC-3′, E_Sarbeco_R: 5′-ATATTGCAGCAGTACGCACACA-3′, wtN_R: 5′-GGTGAACCAAGACGCAGTAT-3′) and probes (E_Sarbeco_P: 5′-FAM-ACACTAGCCATCCTTACTGCGCTTCG-BHQ1-3′, wtN_P: 5′-FAM-TAACCAGAATGGAGAACGCAGTGGG-BHQ1-3′) were used for the RT-qPCR reaction in 384-well plates (Bio-Rad). The total volume of the reaction was 20 μL, and the sample volume was 3.33 μL. CFX384 Touch Real-Time PCR Detection System (Bio-Rad) was used for amplifications. The lower limit of quantification was 50 copies/reaction.

### Statistical analyses

Statistical analyses were performed using non-parametric tests due to small sample size and non-normal data distribution. Paired analyses were used where applicable. All statistical tests were two-tailed and are indicated in the respective figure legends. For comparison of paired samples between two timepoints, Wilcoxon test was used. For comparison of paired samples between three or more timepoints, Friedman’s test with Dunn’s post hoc correction was used. Repeated measures two-way ANOVA with the Geisser-Greenhouse correction and Tukey’s multiple comparisons test was used to compare neutralization of different SARS-CoV-2 variants of longitudinally collected serum samples. Kruskal-Wallis test was used for comparison of non-paired data, with Dunn’s post hoc correction when more than one comparison was performed. Correlation was assessed using Spearman correlation. Kaplan-Meier analysis to evaluate the time until virus clearance between different experimental groups was performed using log-rank test with Bonferroni correction. All statistical tests were performed in Prism v9.4.1.

### Reporting summary

Further information on research design is available in the Nature Research Reporting Summary linked to this article.

### Supplementary information

Supplemental material (figures and tables)

REPORTING SUMMARY

## Data Availability

No unique materials have been generated in this study. Single-cell BCR sequences have been deposited to GenBank under accession numbers OQ993508:OQ994633. Bulk RNA sequencing and single-cell RNA sequencing data have been deposited to NCBI under the BioProject number PRJNA975321.
