# Supplementary material for: Three immunizations with Novavax’s protein vaccines increase antibody breadth and provide durable protection from SARS-CoV-2
Source: NPJ Vaccines. 2024 Jan 20;9:17. doi: 10.1038/s41541-024-00806-2 (PMC10799869; doi:10.1038/s41541-024-00806-2)
Supplement: Supplementary file 1 — Supplemental material (figures and tables) [file 41541_2024_806_MOESM1_ESM.pdf]

**SUPPLEMENTARY INFORMATION**

# Three immunizations with Novavax's protein vaccines increase antibody breadth and provide durable protection from SARS-CoV-2

Klara Lenart, Rodrigo Arcoverde Cerveira, Fredrika Hellgren, Sebastian Ols, Daniel J. Sheward, Changil Kim, Alberto Cagigi, Matthew Gagne, Brandon Davis, Daritza Germosen, Vicky Roy, Galit Alter, Hélène Letscher, Jérôme Van Wassenhove, Wesley Gros, Anne-Sophie Gallouët, Roger Le Grand, Harry Kleanthous, Mimi Guebre-Xabier, Ben Murrell, Nita Patel, Gregory Glenn, Gale Smith, Karin Loré

## SUPPLEMENTARY FIGURES

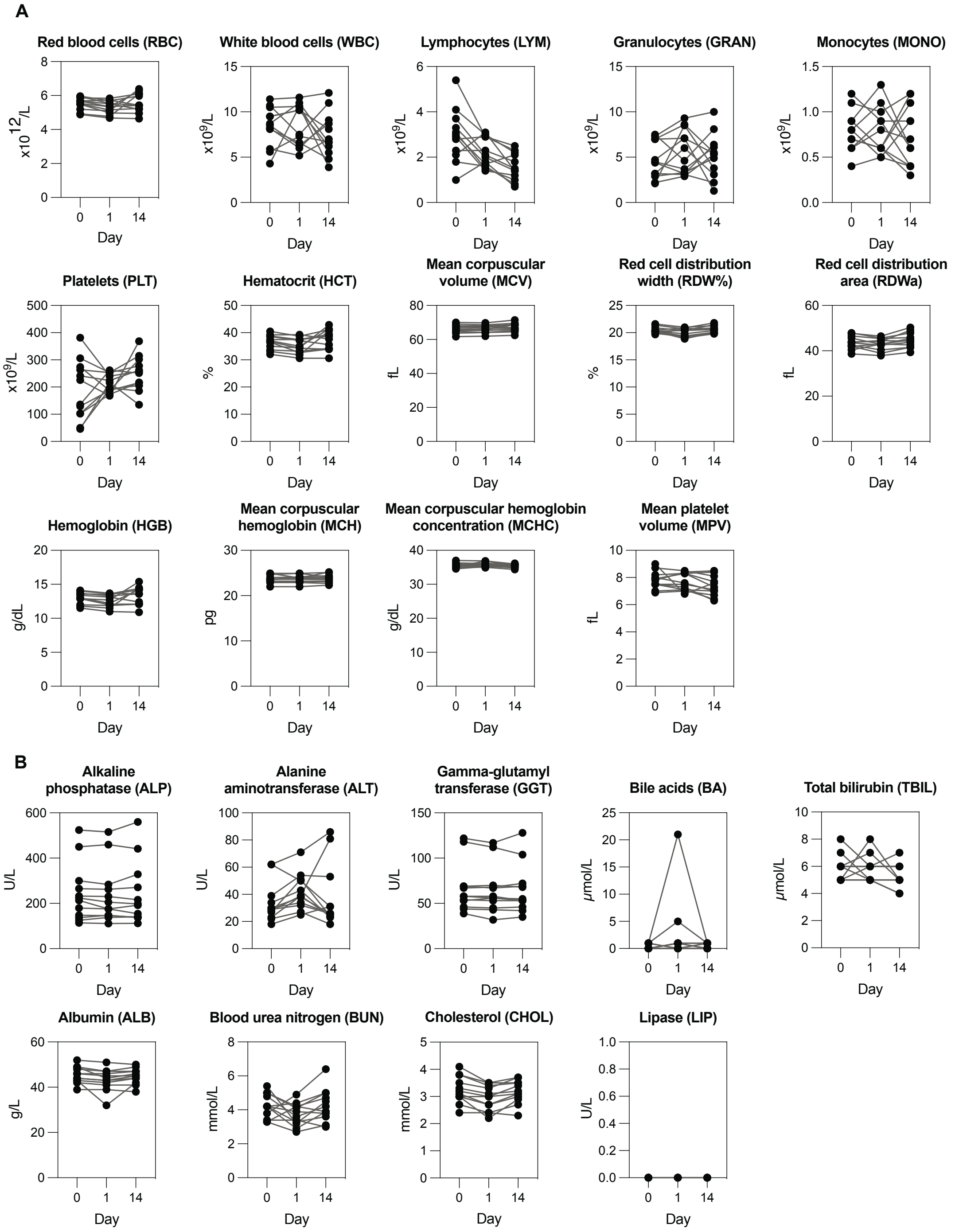


**Supplementary Figure 1: Safety data after Matrix-M immunization.**
(A, B) Clinical hematology (A) and clinical chemistry (B) results at 0, 1 and 14 days after first immunization (n=12).

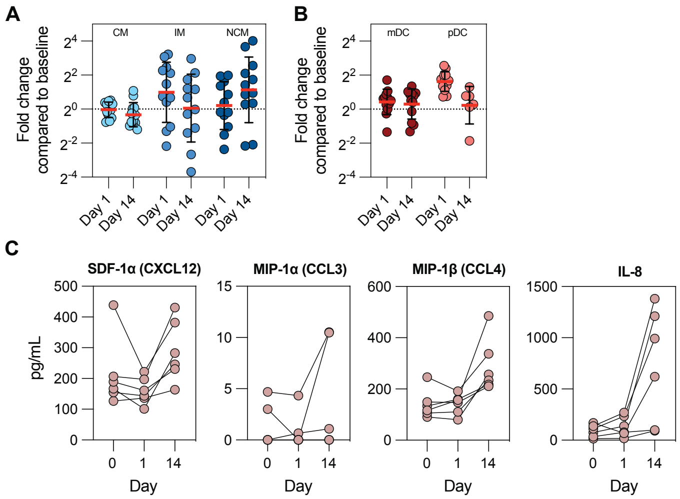


**Supplementary Figure 2: Innate immune responses after Matrix-M immunization.**

(A, B) Fold change in frequency of different monocyte (A) and dendritic cell (B) subsets 1 and 14 days after first immunization compared to day 0 (n=12).

(C) Concentration of selected cytokines in the NHP serum at 0, 1 and 14 days after first immunizations (n=6, related to Figure 1D).

Data is presented as geometric mean ± geometric SD (A, B). The dotted line represents fold change of 1 (i.e. no change from baseline) (A, B).

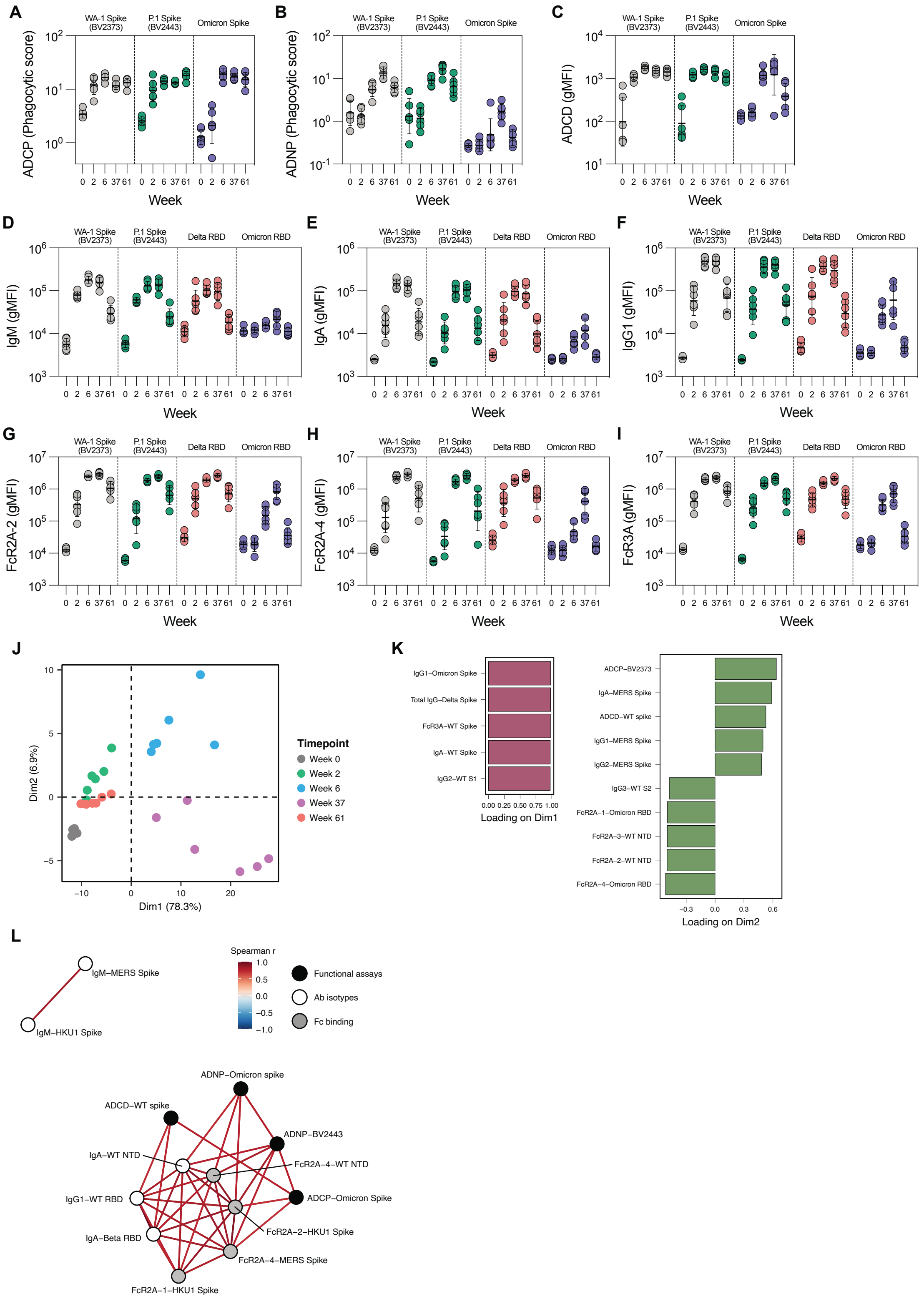


**Supplementary Figure 3: Functional antibody profiling after NVX-CoV2373/CoV2443 immunization.**

(A-C) Vaccine-induced antibody-mediated effector functions for antibody-mediated monocyte phagocytosis (ADCP) (A), antibody-mediated neutrophil phagocytosis (ADNP) (B) and antibody-

mediated complement deposition (ADCD) (C) against WA-1 S (BV2373), P.1 S (BV2443) and B.1.1.529 Omicron S at different timepoints after immunization (n=6).
(D-I) Humoral IgM (D), IgA (E), IgG1 (F) as well as FcR2A-2 (G), FcR2A-4 (H) and FcR3A (I) binding profiles of vaccine-induced antibodies against WA-1 S (BV2373), P.1 S (BV2443), B.1.617.2 Delta RBD and B.1.1.529 Omicron RBD at different timepoints after immunization (n=6).
(J) Principal component analysis (PCA) of antibody features in plasma samples collected at different timepoints after immunization and analyzed by system serology analyses.
(K) Top antibody features influencing loadings on the Dim1 and Dim2 axes of the PCA plot.
(L) Co-correlate network analysis of LASSO-selected antibody features from the systems serology analyses.
Data is presented as geometric mean ± geometric SD (A-I).

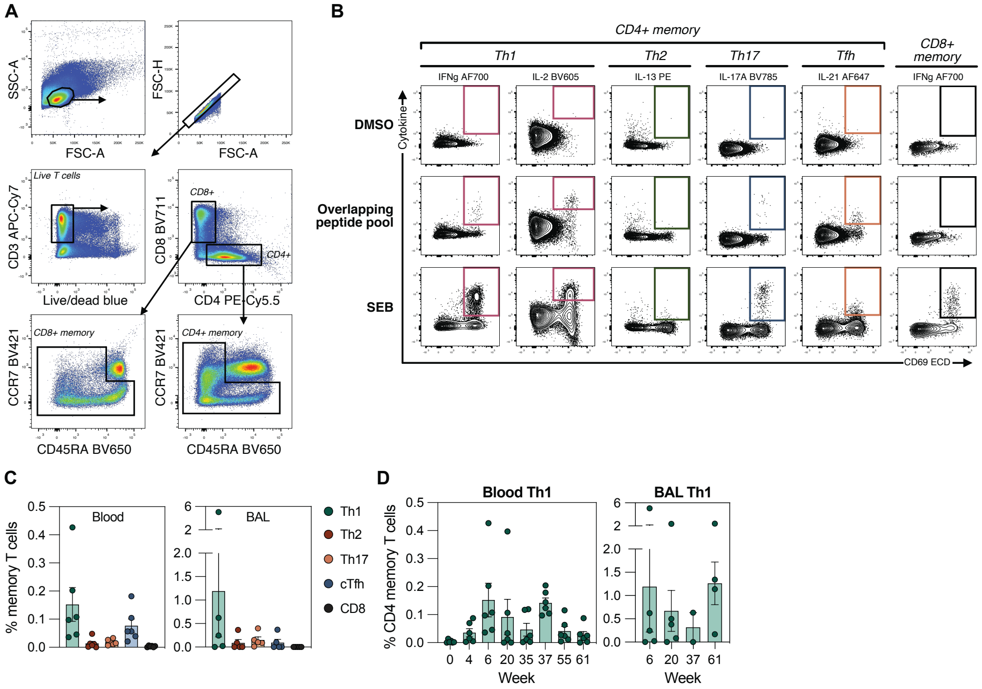


**Supplementary Figure 4: Th1 polarized response in blood and BAL after NVX-CoV2373 immunization.**

(A) Gating strategy for identification of memory T cells.

(B) Representative plots of PBMC samples, stimulated overnight with DMSO, overlapping peptide library of Spike protein, or superantigen SEB in presence of Brefeldin A, for identification of S-reactive memory T cell subsets. All data is background subtracted based on negative control wells, stimulated with DMSO.

(C) Polarization of T cell responses in blood and BAL at week 6, two weeks after boost 1 (n=6).

(D) Kinetics of Th1 memory T cells in blood and BAL (n=6).

Data is presented as mean ± SEM (C-D).

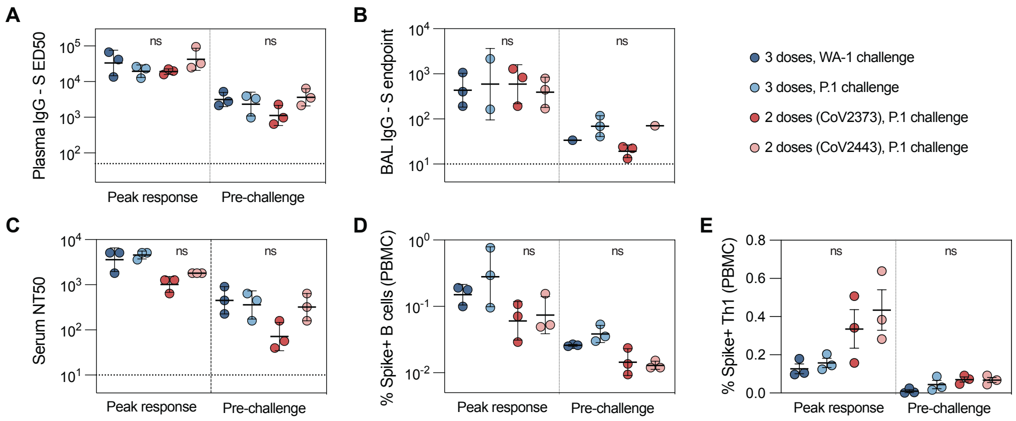


**Supplementary Figure 5: Pre-existing immunity by immunization history.**

(A-E) Pre-existing immunity of vaccinated animals at peak response (two weeks after last immunization) and before challenge: Spike-binding antibodies in serum (A), Spike-binding antibodies in BAL (B), WA-1 neutralizing titers in serum (C), circulating Spike-specific memory B cells (D) and circulating Spike-specific memory Th1 cells (E) (n=3 per group).

Data is presented as geometric mean ± geometric SD (A-E). Statistical analysis was performed using Friedman's test (A, C-E) or Kruskal-Wallis test (B) with Dunn's post hoc correction.

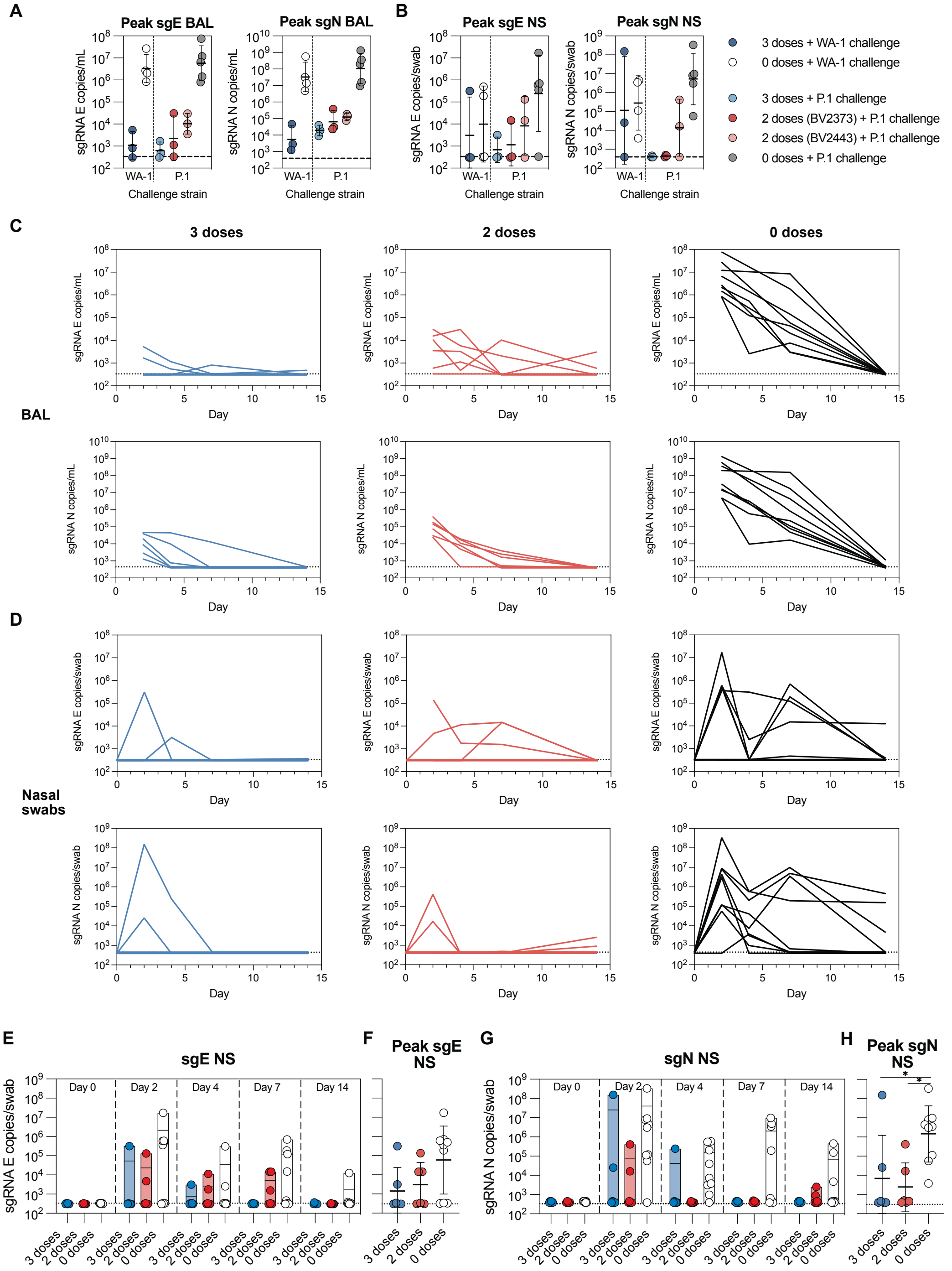


**Supplementary Figure 6: Viral titers in the upper and lower respiratory tract by animal.**

(A-B) Peak sgE and sgN viral loads in BAL (A) and nasal compartment (B) in each subgroup, divided by the immunization regimen and challenge strain (n=3 per group).

(C-D) Detection of viral RNA (sgN and sgE) in lower (BAL) (C) and upper (nasal swabs, NS) (D) respiratory tract. Each line corresponds to a different animal (n=6-9 per group).

(E) Viral loads in NS, divided by number of doses and assessed by sgE gene-targeted RT-PCR (n=6-9 per group).

(F) Peak sgE load in NS of each animal during the 14 day follow-up after challenge (n=6-9 per group).

(G) Viral loads in NS, divided by number of doses and assessed by sgN gene-targeted RT-PCR (n=6-9 per group).
(H) Peak sgN load in NS of each animal during the 14 day follow-up after challenge (n=6-9 per group). Data is presented as mean (min-max) (E, G) or geometric mean ± geometric SD (A, B, F, H). Statistical analysis was performed using Kruskal-Wallis test with Dunn's post hoc correction (F, H).

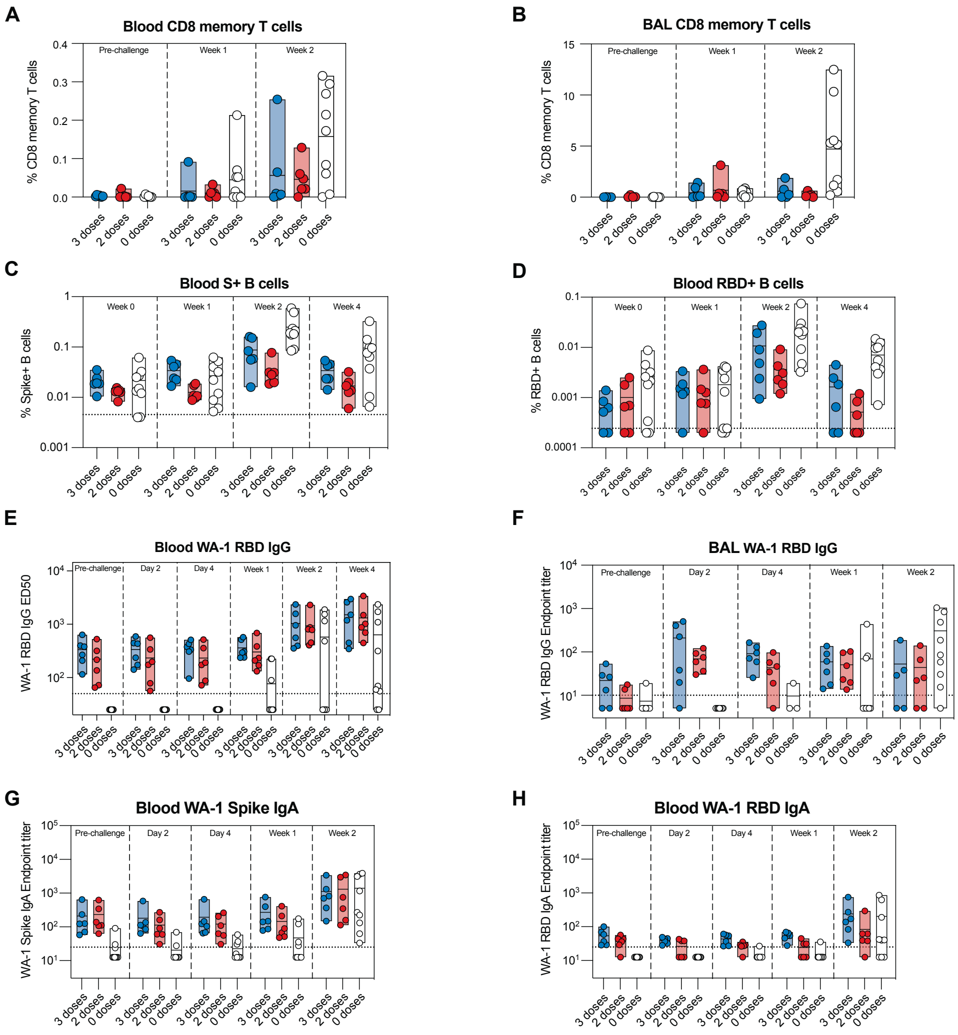


**Supplementary Figure 7: Rapid anamnestic responses in the lungs of immunized animals.**
(A-B) Expansion of Spike-specific CD8 memory T cells in blood (A) and BAL (B) after challenge (n=6-9 per group).
(C-D) WA-1 Spike- (C) and RBD-specific (D) memory B cells in blood after challenge (n=6-9 per group).
(E-F) WA-1 RBD-binding IgG antibodies in blood (E) and BAL (F) after challenge (n=6-9 per group).
(G-H) WA-1 Spike- (G) and RBD-binding (H) IgA antibodies in blood after challenge (n=6-9 per group).
Data is presented as mean (min-max) (A-F). Dotted line corresponds to lower level of detection (C-H).

**SUPPLEMENTARY TABLES**

**Supplementary Table 1: Primer sequences against NHP BCR constant regions, used for 10x library preparation.**

| Primer | Sequence | Master mix |
|---|---|---|
| 10X_RM_IGHA_1 | ACGTGGCATGTCACGGACTC | 1 |
| 10X_RM_IGHD_1 | CTGGCTGCTTGTCGTGTAGCTG | 1 |
| 10X_RM_IGHE_1 | TGTTGACCTCTTTGTCTGCGG | 1 |
| 10X_RM_IGHG_1 | TTGTCCACCTTGGTGTTGCT | 1 |
| 10X_RM_IGHM_1 | TACTTGCCCCCTCTCAGGACT | 1 |
| 10X_RM_IGK_1 | GTCCTGCTCTGTGACACTCTC | 1 |
| 10X_RM_IGL_1 | GTCTCCACTCCCGCGTTGAC | 1 |
| | | |
| 10X_RM_IGHA_2 | TTGCTCCAGGTCACGTTGAGTG | 2 |
| 10X_RM_IGHD_2 | CAGGTGACAGTCACGGACTTTG | 2 |
| 10X_RM_IGHE_2 | GGCTGGTAAGGTCATAGTGCTT | 2 |
| 10X_RM_IGHG_2 | AGCCCTGAGGACTGTAGGA | 2 |
| 10X_RM_IGHM_2 | GCATTCTCACAGGAGACGAGG | 2 |
| 10X_RM_IGK_2 | ATTCAGCAGGCACACAACAGAG | 2 |
| 10X_RM_IGL_2 | AGACACACTAGTGTGGCCTTG | 2 |
