# Supplementary material for: Three immunizations with Novavax’s protein vaccines increase antibody breadth and provide durable protection from SARS-CoV-2
Source: NPJ Vaccines. 2024 Jan 20;9:17. doi: 10.1038/s41541-024-00806-2 (PMC10799869; doi:10.1038/s41541-024-00806-2)
Supplement: Supplementary file 2 — REPORTING SUMMARY [file 41541_2024_806_MOESM2_ESM.pdf]

nature portfolio

Corresponding author(s): Karin Loré

Last updated by author(s): Oct 17, 2023

# Reporting Summary

Nature Portfolio wishes to improve the reproducibility of the work that we publish. This form provides structure for consistency and transparency in reporting. For further information on Nature Portfolio policies, see our Editorial Policies and the Editorial Policy Checklist.

## Statistics

For all statistical analyses, confirm that the following items are present in the figure legend, table legend, main text, or Methods section.

| n/a | Confirmed | |
|---|---|---|
| ☐ | ☒ | The exact sample size (*n*) for each experimental group/condition, given as a discrete number and unit of measurement |
| ☐ | ☒ | A statement on whether measurements were taken from distinct samples or whether the same sample was measured repeatedly |
| ☐ | ☒ | The statistical test(s) used AND whether they are one- or two-sided<br>*Only common tests should be described solely by name; describe more complex techniques in the Methods section.* |
| ☐ | ☒ | A description of all covariates tested |
| ☐ | ☒ | A description of any assumptions or corrections, such as tests of normality and adjustment for multiple comparisons |
| ☐ | ☒ | A full description of the statistical parameters including central tendency (e.g. means) or other basic estimates (e.g. regression coefficient) AND variation (e.g. standard deviation) or associated estimates of uncertainty (e.g. confidence intervals) |
| ☐ | ☒ | For null hypothesis testing, the test statistic (e.g. *F*, *t*, *r*) with confidence intervals, effect sizes, degrees of freedom and *P* value noted<br>*Give P values as exact values whenever suitable.* |
| ☒ | ☐ | For Bayesian analysis, information on the choice of priors and Markov chain Monte Carlo settings |
| ☒ | ☐ | For hierarchical and complex designs, identification of the appropriate level for tests and full reporting of outcomes |
| ☒ | ☐ | Estimates of effect sizes (e.g. Cohen's *d*, Pearson's *r*), indicating how they were calculated |

*Our web collection on statistics for biologists contains articles on many of the points above.*

## Software and code

Policy information about availability of computer code

| | |
|---|---|
| Data collection | Flow cytometry data was collected using commercially available BD FACSDiva software (v8.1 or 9.1, Beckton Dickinson). ELISA data was collected using commercially available SkanIt software (v 7.0, Thermo Fisher). System serology data was collected using IntelliCyt ForeCyt (v 8.1, Sartorius) and CellCapTure 4 (Stratedigm). Luminex data was collected using commercially available Bioplex Manager Software (v 6.2, Bio-Rad). |
| Data analysis | B cell receptor sequences generated via Sanger sequencing were pre-processed with scifer package (v.0.99.3) and aligned using IgDiscover (v.0.15.1). B cell receptor sequences generated using 10x sequencing were processed with CellRanger package (v.3.1.0), Seurat R package (v.4.0.6) and DoubletFinder R package (v.2.0.3), and aligned using IgDiscover (v.0.15.1).<br>Bulk RNA sequening data was processed using nf-core rnaseq pipeline (v.3.7), aligned using STAR alignment (v.2.7.10a) and Salmon package (v.1.8.0), and analyzed using DESeq2 (v.1.34.0) and ClusterProfiler (v.4.2.2).<br>Systems serology analyses were performed using IntelliCyt ForeCyt (v 8.1), FactoMineR R package (v.2.7) and systemsseRology package (available at https://github.com/LoosC/systemsseRology).<br>The 3D model of SARS-CoV-2 Spike protein was edited and colored using UCSD Chimera X (v 1.4).<br>Flow cytometry data analysis was performed using Flowjo (v 10.8.2).<br>Statistical analyses were performed using Graphpad Prism (v.9.0) or R (v.4.2.1) for Mac OS X. |

For manuscripts utilizing custom algorithms or software that are central to the research but not yet described in published literature, software must be made available to editors and reviewers. We strongly encourage code deposition in a community repository (e.g. GitHub). See the Nature Portfolio guidelines for submitting code & software for further information.

## Data

Policy information about availability of data

All manuscripts must include a data availability statement. This statement should provide the following information, where applicable:

- Accession codes, unique identifiers, or web links for publicly available datasets
- A description of any restrictions on data availability
- For clinical datasets or third party data, please ensure that the statement adheres to our policy

Single-cell BCR sequences have been deposited to GenBank under accession numbers OQ993508:OQ994633. Bulk RNA sequencing and single-cell RNA sequencing data has been deposited to NCBI under the BioProject number PRJNA975321. The KIMDB database was used to retrieve the Macaca mulatta germline sequences (Available at: http://kimdb.gkhlab.se/datasets/).

## Research involving human participants, their data, or biological material

Policy information about studies with human participants or human data. See also policy information about sex, gender (identity/presentation), and sexual orientation and race, ethnicity and racism.

| | |
|---|---|
| Reporting on sex and gender | N/A |
| Reporting on race, ethnicity, or other socially relevant groupings | N/A |
| Population characteristics | N/A |
| Recruitment | N/A |
| Ethics oversight | N/A |

Note that full information on the approval of the study protocol must also be provided in the manuscript.

# Field-specific reporting

Please select the one below that is the best fit for your research. If you are not sure, read the appropriate sections before making your selection.

☒ Life sciences ☐ Behavioural & social sciences ☐ Ecological, evolutionary & environmental sciences

For a reference copy of the document with all sections, see nature.com/documents/nr-reporting-summary-flat.pdf

# Life sciences study design

All studies must disclose on these points even when the disclosure is negative.

| | |
|---|---|
| Sample size | The number of non-human primates used was based on the number of animals available and standard practice in the field. |
| Data exclusions | Data points deemed unreliable due to technical experimental issues were excluded in few cases. Bronchoalveolar samples that contained less than 1000 live T cells or total IgG endpoint titer < 1000 were excluded due to suboptimal sampling. |
| Replication | Due to limitations in large animal trials, non-human primate immunization experiments were not replicated. All the samples were run as technical duplicates when possible, this includes all the ELISAs and the neutralization assays performed in this study. |
| Randomization | Animals were not randomly assigned to the groups. The vaccination grouping was done controlling for sex and weight in order to have similar distribution of these factors between the study groups. The control group in the challenge experiment was not perfectly matched due to limited availability of naive NHPs during the pandemic. |
| Blinding | Investigators were not blinded to data collection and/or data analysis due to lack of personnel and resources. |

# Reporting for specific materials, systems and methods

We require information from authors about some types of materials, experimental systems and methods used in many studies. Here, indicate whether each material, system or method listed is relevant to your study. If you are not sure if a list item applies to your research, read the appropriate section before selecting a response.

## Materials & experimental systems

| n/a | Involved in the study |
| --- | --- |
| ☐ | ☒ Antibodies |
| ☒ | ☐ Eukaryotic cell lines |
| ☒ | ☐ Palaeontology and archaeology |
| ☐ | ☒ Animals and other organisms |
| ☒ | ☐ Clinical data |
| ☒ | ☐ Dual use research of concern |
| ☒ | ☐ Plants |

## Methods

| n/a | Involved in the study |
| --- | --- |
| ☒ | ☐ ChIP-seq |
| ☐ | ☒ Flow cytometry |
| ☒ | ☐ MRI-based neuroimaging |

# Antibodies

| | |
| --- | --- |
| Antibodies used | Information about all the antibodies used in the flow cytometry panels and ELISAs are described in their corresponding methods' sections. |
| Validation | Reactivity of all antibody reagents used in this study were based on manufacturer's reported information on manufacturer's website. |

# Animals and other research organisms

Policy information about studies involving animals; ARRIVE guidelines recommended for reporting animal research, and Sex and Gender in Research

| | |
| --- | --- |
| Laboratory animals | Fifteen Indian rhesus macaques (Macaca mulatta) of approximate 4-6 years of age and six Chinese rhesus macaques (Macaca mulatta) of approximate 3-4 years of age were used in this study.<br>Biopsies for RNA sequencing were collected as a part of a published study (Ols et al, PMID: 32209459). Biopsies from two Chinese rhesus macaques (Macaca mulatta) of approximately 4-5 years of age were used. |
| Wild animals | This study did not involve wild animals. |
| Reporting on sex | Fifteen Indian rhesus macaques (Macaca mulatta), seven females and eight males, and six Chinese rhesus macaques (Macaca mulatta), all males, were used. For immunizations, twelve Indian rhesus macaques, six females and six males, were divided into groups with similar distribution of age, sex and weight. Six male Chinese rhesus macaques, two male and one female Indian rhesus macaques were included as control animals in the challenge experiment.<br>Two female Chinese rhesus macaques were part of the study where local effects of Matrix-M and Matrix-M adjuvanted vaccines were studied, and where we collected tissue biopsies used for RNA sequencing in this study. |
| Field-collected samples | This study did not involve field-collected samples. |
| Ethics oversight | This study was approved by the Stockholm Regional Ethical Board on Animal Experiments (16344-2017, 18427-2019), institutional ethical committee “Comité d’Ethique en Expérimentation Animale du Commissariat à l’Energie Atomique et aux Energies Alternatives” and the French Ministry of Higher Education and Research (A20_061). |

Note that full information on the approval of the study protocol must also be provided in the manuscript.

# Flow Cytometry

## Plots

Confirm that:

☒ The axis labels state the marker and fluorochrome used (e.g. CD4-FITC).

☒ The axis scales are clearly visible. Include numbers along axes only for bottom left plot of group (a 'group' is an analysis of identical markers).

☒ All plots are contour plots with outliers or pseudocolor plots.

☒ A numerical value for number of cells or percentage (with statistics) is provided.

## Methodology

| | |
| --- | --- |
| Sample preparation | PBMC were isolated using a Ficoll density gradient protocol and either cryopreserved or used directly. BAL cells were washed with PBS, filtered through 70um cell strainer before use and used fresh. PBMCs were stained with fluorescently labeled antibodies for analysis of innate immune cells and memory B cells. For T cell assays, PBMCs and BAL cells were stimulated with antigenic peptides as described in the methods section and then stained with a panel of fluorescent antibodies for analysis of intracellular cytokines. |
| Instrument | Samples were acquired on a BD LSRFortessa flow cytometer. The exception were samples at weeks 6 and 37, when |

| | |
|---|---|
| Instrument | acquisition was performed on a BD FACSAria Fusion instrument in order to combine data acquisition with simultaneous single-cell sorting into 96-well PCR plates or sorting of Spike-specific population for 10x sequencing. |
| Software | Data was acquired with BD FACSDiva (Beckton Dickinson) version 8.0.1 or 9.0.1, and analyzed using Flowjo version 10 (BD Life Sciences). |
| Cell population abundance | Single-cell index sorts were performed for the isolation of Spike-specific memory B cells. For 10x sequencing, purity of samples was not determined post sort due to extremely limited yied. |
| Gating strategy | Details regarding the gating strategy for each of the subset studied is described in detail in the supplementary material with representative figures.<br>In the innate panel, neutrophils were identified as live single CD66abce+ SSC high cells, T cells were identified as live single CD66abce- CD3+, B cells as live single CD66abce- CD20+ cells and natural killer (NK) cells as live single CD66abce- CD3- CD20- HLA-DR- NKg2A+ cells. Monocytes were first defined as live single CD66abce- CD3- CD20- NKg2A- HLA-DR+ cells, and this population was further divided in monocyte subsets: classical monocytes as CD14+ CD16-, intermediate monocytes as CD14+ CD16+ and non-classical monocytes as CD14- CD16+. Dendritic cell subsets were defined out of CD14- CD16- double negative population: myeloid DCs as CD123- CD11c+ and plasmacytoid DCs as CD11c- CD123+.<br>Memory B cells were defined as live single CD3- CD11c- CD14- CD16- CD123- CD20+ HLA-DR+ IgM- IgG+ cells, out of which we defined Spike-specific memory B cells as cells that are double positive for the fluorescently labelled Spike probes in two colors (APC and PE). Within the Spike-specific population, the B cells that also bound fluorescently labelled RBD were defined as RBD-specific.<br>CD4/CD8+ memory T cells were first identified as live single CD3+ CD8- CD4+ NOT(CD45RA+CCR7+) cells for CD4 memory and live single CD3+ CD4- CD8+ NOT(CD45RA+CCR7+) cells for CD8 memory. Then, activated CD69+ cells were analyzed for the presence of different intracellular cytokines; IFNg, IL-2, IL-17A, IL-13 and IL-21. |

☒ Tick this box to confirm that a figure exemplifying the gating strategy is provided in the Supplementary Information.
